# MicroRNA binding site variation is enriched in psychiatric disorders

**DOI:** 10.1002/humu.24481

**Published:** 2022-10-23

**Authors:** Michael P. Geaghan, William R. Reay, Murray J. Cairns

**Affiliations:** ^1^ School of Biomedical Sciences and Pharmacy The University of Newcastle Callaghan New South Wales Australia; ^2^ Precision Medicine Research Program Hunter Medical Research Institute New Lambton Heights New South Wales Australia

**Keywords:** common variants, MBSV, microRNA, microRNA binding site, MRE, psychiatric disorders

## Abstract

Psychiatric disorders have a polygenic architecture, often associated with dozens or hundreds of independent genomic loci. Most associated loci impact noncoding regions of the genome, suggesting that the majority of disease heritability originates from the disruption of regulatory sequences. While most research has focused on variants that modify regulatory DNA elements, those affecting *cis*‐acting RNA sequences, such as miRNA binding sites, are also likely to have a significant impact. We intersected genome‐wide association study (GWAS) summary statistics with the dbMTS database of predictions for miRNA binding site variants (MBSVs). We compared the distributions of MBSV association statistics to non‐MBSVs within brain‐expressed 3′UTR regions. We aggregated GWAS *p* values at the gene, pathway, and miRNA family levels to investigate cellular functions and miRNA families strongly associated with each trait. We performed these analyses in several psychiatric disorders as well as nonpsychiatric traits for comparison. We observed significant enrichment of MBSVs in schizophrenia, depression, bipolar disorder, and anorexia nervosa, particularly in genes targeted by several miRNA families, including miR‐335‐5p, miR‐21‐5p/590‐5p, miR‐361‐5p, and miR‐557, and a nominally significant association between miR‐323b‐3p MBSVs and schizophrenia risk. We identified evidence for the association between MBSVs in synaptic gene sets in schizophrenia and bipolar disorder. We also observed a significant association of MBSVs in other complex traits including type 2 diabetes. These observations support the role of miRNA in the pathophysiology of psychiatric disorders and suggest that MBSVs are an important class of regulatory variants that have functional implications for many disorders, as well as other complex human traits.

## 1 INTRODUCTION

Psychiatric disorders, including schizophrenia (SCZ), major depressive disorder (MDD), bipolar disorder (BIP), and autism spectrum disorders (ASD) are prevalent to varying degrees in the general population (approximately 1% for SCZ, BIP, and autism; approximately 35% for MDD), and are responsible for considerable morbidity and mortality. Existing treatments have limited efficacy; for example, in the case of antipsychotics, there is a 27% 1‐year relapse rate (Leucht et al., 2012) and a range of detrimental side effects, such as cardiometabolic dysfunction (Lally & MacCabe, 2015) and other complications, which can reduce compliance. These drawbacks are partly due to a focus on symptoms, due in turn to a severe lack of understanding of the etiological origins of these disorders.

In recent years, significant focus in the context of psychiatric disease has been given to a class of small, noncoding RNAs known as microRNAs (miRNAs) (M. Geaghan & Cairns, 2015). MiRNAs are important regulators of gene expression and translation. They recognize and bind to target mRNA molecules, typically within the 3′UTR region via partial complementary binding to miRNA recognition elements (MREs). This complementary binding occurs primarily between the mRNA and the nucleotides 2–8 of the miRNA, referred to as the “seed region.” Active miRNA are associated with a protein complex known as the RNA‐Induced Silencing Complex (RISC), which directs either silencing of protein translation or degradation of the mRNA molecule, thus resulting in posttranscriptional regulation of gene expression (Bartel, 2004). The short recognition sequence of mammalian miRNAs means that a single miRNA sequence is capable of targeting numerous genes, typically within the hundreds. Thus, miRNA–mRNA interaction networks are complex and differ from cell to cell and from tissue to tissue, dependent upon the local gene and miRNA expression profiles. While this complexity makes target prediction for miRNAs difficult, several algorithms have been developed to make credible bioinformatic predictions of the targets of miRNAs.

There is mounting evidence from both genetics and gene expression studies supporting a role for genetic and environmental alteration of miRNA function in psychiatric disease; indeed, the genetic locus containing the miR‐137 host gene *MIR137HG* is host to one of the most significant common genetic associations with SCZ (Pardiñas et al., 2018; Schizophrenia Working Group of the Psychiatric Genomics Consortium, 2014). What has been less thoroughly studied is the possibility for an accumulation of genetic variants present within the MREs of genes involved in psychiatrically relevant networks. In the present study, we identified miRNA binding site variants (MBSVs) and putative gene pathways affected by these variants in nine psychiatric disorders with existing genome‐wide association study (GWAS) summary data: SCZ, BIP, MDD, ASD, posttraumatic stress disorder (PTSD), attention deficit hyperactivity disorder (ADHD), anorexia nervosa (AN), obsessive compulsive disorder (OCD), and Tourette's syndrome (TS). These analyses revealed a significant enrichment of MBSVs in several disorders, including SCZ, BIP, MDD, and AN. We also found evidence for the aggregation of MBSVs within pathways relevant to synaptic function. These results suggest that MBSVs may contribute to the etiology and/or pathophysiology of disorders such as SCZ and BIP, which suggests these may be valuable targets for future research into disease origins and novel treatments.

## 2 MATERIALS AND METHODS

### 2.1 GWAS data

GWAS summary statistics were obtained from the Psychiatric Genomics Consortium website (https://www.med.unc.edu/pgc/download-results/) for the following nine psychiatric disorders: BIP, MDD, ASD, PTSD, ADHD, AN, OCD, and TS. For SCZ, the summary statistics from the more recent 2018 meta‐analysis performed by the CLOZUK consortium (Pardiñas et al., 2018) were obtained (https://walters.psycm.cf.ac.uk/); similarly, for each PGC GWAS, the latest available summary statistics were used: BIP—2018 (Stahl et al., 2019); MDD—2019 (Howard et al., 2019); ASD—2019 (Grove et al., 2019); PTSD—2019 (Nievergelt et al., 2019); ADHD—2018 (Demontis et al., 2019); AN—2019 (Watson et al., 2019); OCD—2018 (International Obsessive Compulsive Disorder Foundation Genetics Collaborative [OCDF‐GC] and OCD Collaborative Genetics Association Studies [OCGAS], 2018); TS—2019 (Yu et al., 2019). A summary of the GWAS data is presented in Table 1.

**Table 1:** Summary of summary statistics and numbers of variants and MBSVs identified in each psychiatric GWAS

GWAS	# Variants	# Pruned variants	*h* ^2^_SNP_	Cases/controls
*GWAS variants*				
ADHD	704,4977	391,333	0.22	19,099/34,194
AN	7,205,595	414,559	0.11	16,992/55,525
ASD	7,920,272	622,935	0.12	18,381/27,969
BIP	11,007,695	835,573	0.17	20,352/31,358
MDD	8,377,213	707,808	0.089 (0.06)	170,756/329,443
OCD	8,100,892	674,944	0.23	2688/7037
PTSD	8,501,767	791,574	0.04	23,212/151,447
SCZ	7,487,369	594,558	0.23	40,675/64,643
TS	8,017,356	683,821	0.21	4819/9488

GWAS	**# Variants (|diffScore|**≥**0.2)**	**# Pruned variants (|diffScore|**≥**0.2)**	**# Variants (|diffScore|**≥ **0)**	**# Pruned variants (|diffScore|**≥ **0)**
*MBSVs*				
ADHD	4503	3123	5215	3163
AN	4640	3244	5374	3288
ASD	5157	3617	5977	3686
BIP	7929	3995	9189	4086
MDD	5753	3746	6659	3823
OCD	5443	3757	6333	3833
PTSD	5576	3918	6477	4004
SCZ	4916	3502	5700	3562
TS	5446	3788	6324	3868
*GTEx eQTL MBSVs*				
ADHD	1595	1021	1854	1027
AN	1610	1030	1871	1037
ASD	1633	1046	1898	1054
BIP	1673	1057	1948	1066
MDD	1625	1035	1886	1043
OCD	1648	1056	1915	1064
PTSD	1654	1057	1924	1065
SCZ	1594	1023	1855	1030
TS	1646	1052	1908	1060
*GTEx Brain eQTL MBSVs*				
ADHD	362	210	430	211
AN	361	210	427	211
ASD	370	217	438	218
BIP	371	217	439	218
MDD	362	212	429	213
OCD	369	217	434	218
PTSD	371	217	439	218
SCZ	359	212	427	213
TS	368	216	433	217
*PSYCHENCODE eQTL MBSVs*				
ADHD	321	192	369	192
AN	326	194	377	194
ASD	333	200	386	200
BIP	348	208	406	208
MDD	337	202	392	202
OCD	342	207	397	207
PTSD	343	209	398	209
SCZ	328	197	379	197
TS	339	205	393	205

Abbreviations: ADHD, attention deficit hyperactivity disorder; AN, anorexia nervosa; ASD, autism spectrum disorders; BIP, bipolar disorder; GWAS, genome‐wide association study; MBSV, miRNA binding site variant; MDD, major depressive disorder; OCD, obsessive compulsive disorder; PTSD, posttraumatic stress disorder; SCZ, schizophrenia; TS, Tourette's syndrome.

### 2.2 Identification of MBSVs

To identify all potential common MBSVs associated with psychiatric disorders, we first took the union of all single nucleotide variants (SNVs) assessed in all GWAS. The chromosome, position, reference genome allele and alternate allele for each variant was identified. This information was then used to query the dbMTS database (v1.0) (C. Li et al., 2020)—an extension of the dbNSFP database containing all putative SNVs predicted to affect MREs based on three separate target prediction algorithms (TargetScan v7.0 [Lewis et al., 2005], miRanda [August 2010 release] [Enright et al., 2003; John et al., 2004], and RNAhybrid v2.1.1 [Rehmsmeier et al., 2004]).

#### 2.2.1 Identification of brain‐expressed genes and miRNAs

Both mRNA and miRNA brain expression data were obtained from the BrainSpan Atlas Developmental Transcriptome RNA sequencing data set (v10) (J. A. Miller et al., 2014). These data were filtered to remove sequencing data from individuals less than 13 years of age, and represented samples from 16 brain regions, including the dorsolateral prefrontal cortex (DFC), ventrolateral prefrontal cortex (VFC), anterior cingulate cortex (MFC), orbital frontal cortex (OFC), primary motor cortex (M1C), primary somatosensory cortex (S1C), posteroinferior parietal cortex (IPC), primary auditory cortex (A1C), posterior superior temporal cortex (STC), inferolateral temporal cortex (ITC), primary visual cortex (V1C), hippocampus (HIP), amygdaloid complex (AMY), striatum (STR), mediodorsal nucleus of thalamus (MD), and cerebellar cortex (CBC). The mRNA data was available as RPKM‐normalized values. Genes were filtered for a minimum RPKM of 1 across at least 75% of samples. MiRNA data was available as raw read counts. These data were normalized to counts‐per‐million (CPM) by dividing the raw read counts by the total read count (in millions) of the respective samples. Then, the miRNAs were filtered for a minimum CPM of 10.30 (equivalent to a raw count of 10 in the smallest library) across 75% of the samples. The miRNAs were further filtered for confidently‐annotated miRNA species, as determined by the TargetScan database (v7.2). This includes miRNAs annotated by TargetScan as “highly conserved,” “conserved” and “poorly conserved but confidently annotated,” and excludes the “poorly conserved and possibly misannotated as a miRNA” category.

#### 2.2.2 Variant filtering

Nonautosomal variants were filtered out before any analysis. Variants were further filtered by the expression of the affected miRNAs and mRNAs within the brain. Next, information on both the reference and alternate alleles for each variant was extracted from the dbMTS data. Alleles were considered to overlap a “true” MRE if both TargetScan and at least one of the other two algorithms annotated it for the same miRNA and gene. For each surviving allele, the TargetScan predictions were then used for all remaining analyses. Variants within the major histocompatibility (MHC) region on chromosome 6 (positions 28,477,797–33,448,354, build hg19) were excluded due to the genomic complexity within the region.

#### 2.2.3 Calculation of variant scores

For each allele‐miRNA‐transcript combination, the dbMTS data contained a target prediction score calculated by TargetScan. For each gene, the best (i.e., most negative) score for each allele‐miRNA combination across all transcripts was retained as a representative score for that allele‐miRNA–gene interaction. Next, a representative “difference score” for each variant‐gene interaction was calculated by subtracting the best reference allele score from the best alternate allele score. For the subset of variants that affected the 3′UTRs of multiple genes, the best difference score was retained as the representative score for that variant. As such, each score represented the effect a variant had on the optimal miRNA binding conditions. A positive difference score represented a loss of miRNA binding affinity, and a negative score represented a gain, reflecting the negative scoring system of TargetScan. Furthermore, for many of the downstream analyses, “true” MBSVs were considered to be those with an absolute difference score ≥0.2, which equated to removing the lower 15% of scores.

### 2.3 Annotation of eQTLs

The dbMTS data contained eQTL information for each variant obtained from the GTEx database v6 (Lonsdale et al., 2013), including each gene and respective tissue for which the variant was an eQTL. MBSVs were considered an eQTL if they affected the same gene that was annotated in the GTEx data. Furthermore, MBSVs were considered brain‐eQTLs if they affected the same gene in any brain tissue. Additionally, variants were cross‐referenced with brain‐eQTLs identified by the PSYCHENCODE consortium (D. Wang et al., 2018). PSYCHENCODE eQTLs were filtered by an FDR < 0.05 and an fragments per kilobase million > 0.1 in at least 150 samples.

### 2.4 Annotation of CADD scores

We annotated all GWAS variants, including MBSVs for CADD (combined annotation‐dependent depletion) scores (Rentzsch et al., 2019). CADD scores were obtained directly from the CADD SNV database, using both the latest available version (v1.6) in addition to the original published version (v1.0) (Kircher et al., 2014). The original database was used to determine if CADD scores were biased for MBSVs due to the inclusion of miRNA target prediction score annotations in the generation of the scores from v1.1 onward.

### 2.5 Association of MBSVs with psychiatric disorders

To assess whether MBSVs were associated with psychiatric disorders, we first investigated the empirical cumulative distribution functions (ECDFs) of *p* values associated with MBSVs in each disorder and compared these to those of all non‐MBSV variants located within brain‐expressed 3′UTR regions. ECDFs for eQTL subsets of MBSVs were also calculated. Variants were first pruned using PLINK (v1.90) using the European subset of the 1000 Genomes Phase 3 cohort (*N* = 503), a window and step size of 50 and 5 nucleotides, respectively, a minor allele frequency (MAF) threshold of 0.01 and an *R* ^2^ threshold of 0.1. When pruning, MBSVs were prioritized. ECDFs were compared using the Kolmogorov–Smirnov (K–S) test. ECDFs of the absolute log effect sizes (i.e., log odds ratio [OR] or *β*) were also calculated and compared. Furthermore, we calculated ECDFs for CADD score ranks (v1.6 and v1.0) of all MBSVs and 3′UTR non‐MBSVs across all nine GWAS and compared these with the K–S test. We next conducted quantile regression on the negative log_10_‐transformed *p* values and absolute log effect sizes at four quantiles: the median, 90th percentile, 99th percentile, and 99.9th percentile. The regressions were modeled as outcome~variant class, where the outcome was either −log_10_ *p* value or absolute log effect size, and the variant class was a binary variable with 0 equal to the non‐MBSV 3′UTR class and 1 equal to one of either: all MBSVs; GTEx eQTL MBSVs; GTEx brain eQTL MBSVs; or PSYCHENCODE MBSVs. Thus, the variant class coefficient represented the effect of MBSVs relative to non‐MBSVs. Standard errors and significance were calculated using the bootstrap method with 200 replications. We also conducted median (quantile) regression for CADD score ranks of all MBSVs and 3′UTR non‐MBSVs across all nine GWAS in a similar manner.

For each disorder, the proportion of MBSVs that were genome‐wide significant (*p* < 5 × 10^−8^) and suggestive‐significant (*p* < 1 × 10^−5^) was compared to the proportion of either all non‐MBSV variants or non‐MBSV 3′UTR variants, using Fisher's exact test to assess whether there was any enrichment or depletion of MBSVs. For both K–S and Fisher's exact tests, the false discovery rate (FDR) across all tests for each disorder was calculated using the Benjamini–Hochberg method, and an FDR < 0.05 was considered significant.

We next aggregated MBSV *p* values obtained from the respective GWAS summary statistics, in a manner analogous to traditional gene‐set association analysis. To account for the correlation between *p* values for MBSVs in linkage disequilibrium (LD), we applied the aggregated Cauchy association test (ACAT) method (Liu et al., 2019). The advantage of this method is its computational simplicity and its low sensitivity to correlated *p* values, particularly when the aggregated *p* value is small. This method takes *p* values $p_{i}$ for i=(0,1,…,k) and optional non‐negative weights $w_{i}$ and returns an approximate *p* value $p_{\mathrm{ACAT}}$ representing the aggregated association:

$$T_{\mathrm{ACAT}}=\sum_{i=1}^{k}w_{i}\tan\left[\left(0.5-p_{i}\right)\pi \right],$$

$$w=\sum_{i=1}^{k}w_{i},$$

$$p_{\mathrm{ACAT}}\approx \frac{1}{2}-\frac{\arctan\left(T_{\mathrm{ACAT}}/w\right)}{\pi }.$$

For each psychiatric disorder, we used the GWAS *p* values for all MBSVs and weighed them by the absolute difference score. To further dissect the role of MBSVs in psychiatric disorders, we repeated the ACAT *p* value aggregation on subsets of variants corresponding to each miRNA family the variants affected. In each case, four tests were conducted per GWAS/meta‐analysis: all MBSVs, GTEx eQTL MBSVs, GTEx brain eQTL MBSVs, and PSYCHENCODE eQTL MBSVs. Significance was determined using a Bonferroni‐corrected *p* value across all ACAT tests for all disorders.

### 2.6 Gene‐ and gene set‐level analysis of MBSV‐affected pathways associated with psychiatric disorders

To investigate whether MBSVs were significantly aggregated within gene ontology gene sets associated with each disorder, we used MAGMA (v1.07) (de Leeuw et al., 2015). MBSVs were used to filter the GWAS variants supplied to MAGMA. Gene‐level analyses and competitive gene set association tests were performed using gene ontology sets. Genes with a Bonferroni‐corrected *p* < 0.05 were considered significant. Due to gene ontology sets not being strictly independent of one another, a relaxed significance threshold of an FDR‐corrected *p* < 0.05 was applied to the gene set‐level analyses. Gene set association was tested for all MBSVs, as well as MBSVs annotated as either GTEx eQTLs, GTEx brain eQTLs or PSYCHENCODE eQTLs.

### 2.7 Meta‐analysis of MBSV and MBSV target pathway associations across psychiatric disorders

To investigate whether there were general associations between MBSV‐affected miRNA families and gene pathways across all nine disorders, we applied a meta‐analysis technique. For the gene‐set association meta‐analysis, we used the meta option in MAGMA. This option utilizes Stouffer's weighted *Z*‐score meta‐analysis method. Briefly, *p* values are converted to a standardized *Z*‐score via the probit function. The *Z*‐scores are then subsequently combined:

$$Z_{i}=\Phi^{-1}\left(1-p_{i}\right),$$

$$Z_{\mathrm{meta}}=\frac{\sum_{i=1}^{N}w_{i}Z_{i}}{\sqrt{\sum_{i=1}^{N}w_{i}^{2}}},$$

$$p_{\mathrm{meta}}=1-\Phi \left(Z_{\mathrm{meta}}\right).$$

The *Z*‐scores weights $w_{i}$ are the sample sizes of the respective GWAS. We further employed this same method to meta‐analyse the ACAT‐generated *p* values for the miRNA family associations.

### 2.8 Analysis of difference score associations with GWAS effect size

To determine whether any specific miRNA families demonstrated a significant relationship between the difference score of each MBSV and the associated GWAS effect size for each disorder, we conducted a series of linear regression analyses. Effect sizes were standardized to be relative to the alternate allele by inverting the sign of the log‐transformed values if the original GWAS data was calculated relative to the reference allele. We additionally investigated the relationship between the sign of the difference scores and effect sizes. For each disorder, we only investigated those miRNA families which showed a significant ACAT‐aggregated *p* value.

### 2.9 Comparison against nonpsychiatric traits

To determine whether our findings were specific to psychiatric disorders or a more general phenomenon of human traits, we repeated the above analyses for several nonpsychiatric traits, including body mass index (BMI) (Pulit et al., 2019), type 2 diabetes (both adjusted and unadjusted for BMI) (Mahajan et al., 2018), height (Yengo et al., 2018), and cardiovascular disease (CAD) (EPIC‐CVD Consortium, CARDIoGRAMplusC4D, The UK Biobank CardioMetabolic Consortium CHD working group et al., 2017). To identify MBSVs for these traits, we used blood gene and miRNA expression as a reference; we further repeated the type 2 diabetes analyses using the pancreas gene and miRNA expression as a reference. Similarly, in eQTL analyses, MBSVs were filtered for those annotated in the respective tissues in the GTEx database. Blood gene and miRNA expression data were acquired from RNA sequencing of peripheral blood mononuclear cells (PBMCs) obtained from 15 healthy individuals in the Australian Schizophrenia Research Bank (ASRB) as detailed in a previous publication (M. P. Geaghan et al., 2019). Both gene and miRNA expression were filtered for a minimum CPM value of 0.381 and 9.21, respectively, in at least 75% of the samples, equating to a minimum count of 10 reads in the smallest libraries. Pancreas tissue expression data was obtained from publicly released sequencing data of five healthy individuals as part of a study on pancreatic cancer (Müller et al., 2015). These data were available as averaged normalized expression values (NEV), and were filtered for a NEV greater than 10.

### 2.10 Code availability

All R and bash scripts used for these analyses, as well as software version information is available at the following GitHub repository: https://github.com/mgeaghan/mbsv.

## 3 RESULTS

### 3.1 MBSVs are enriched for higher functional scores

Utilizing the dbMTS database, we investigated the union of all variants tested in each of the nine psychiatric disorder GWAS. From the list of allele‐miRNA–mRNA interactions obtained from the database, we identified those interactions predicted by TargetScan and at least one of the other two algorithms (RNAhybrid and miRanda). We calculated scores representing the difference between the best reference allele interactions and the best alternate allele interactions and discounted variants with small scores (absolute difference score < 0.2). In total, we identified a total of 8334 variants, which we considered to be “true” MBSVs. On average, MBSVs accounted for ~0.067% of the SNVs in each GWAS (SD = 0.0025%), with between 3123 and 3995 independent (pruned) MBSVs out of 391,333–835,573 total variants (mean proportion of independent MBSVs = 0.59%, SD = 0.12%) (Table 1).

**Figure 1:**
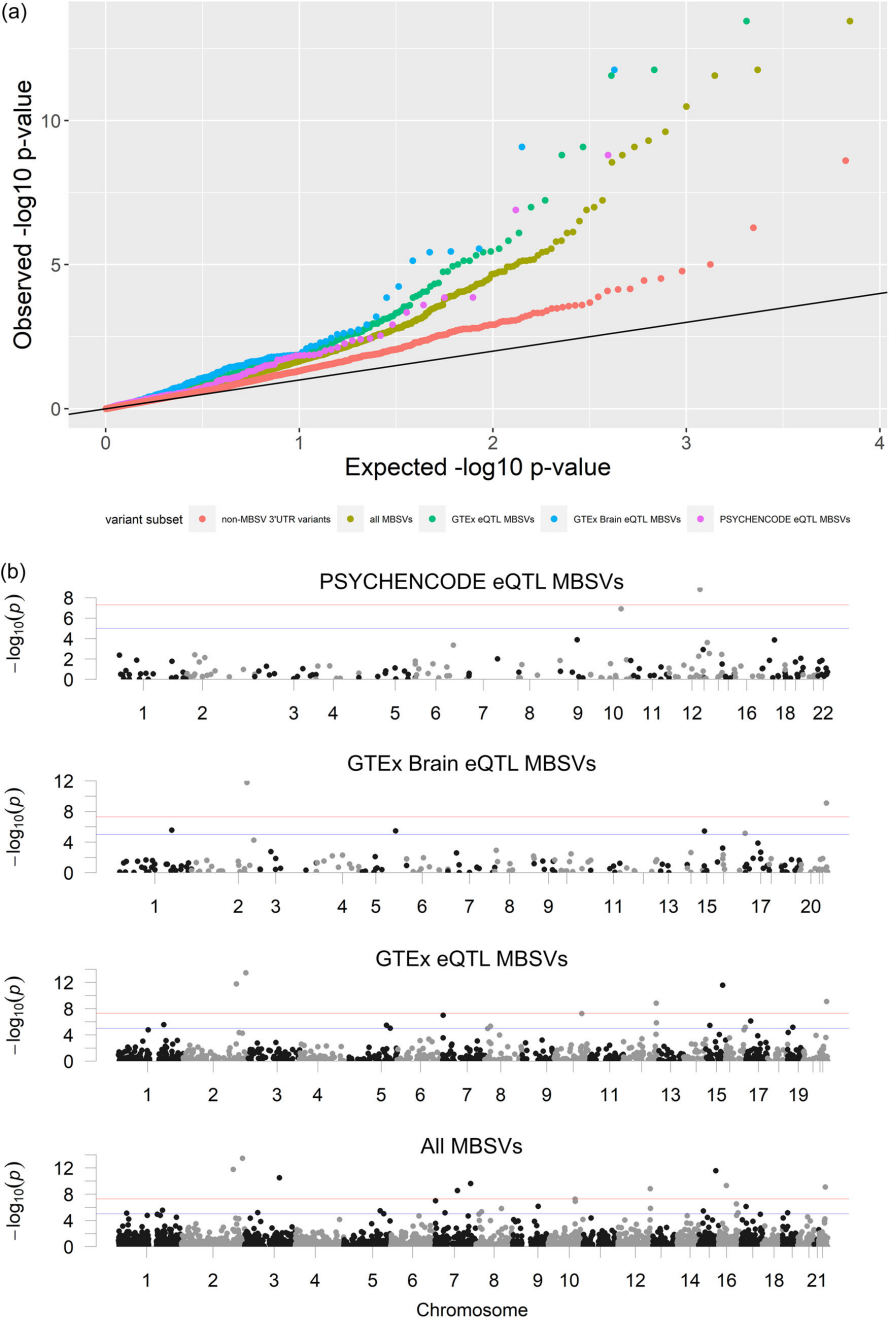
(a) Quantile‐quantile plot of negative log_10_ *p* values of all non‐MBSVs located in brain‐expressed 3′UTR regions (red), all MBSVs (gold), GTEx eQTL MBSVs (green), GTEx brain eQTL MBSVs (blue) and PSYCHENCODE MBSVs (purple) in SCZ. (b) Manhattan plots of the distribution of MBSVs and eQTL MBSV subsets across the genome. MBSV, miRNA binding site variant; SCZ, schizophrenia.

**Figure 2:**
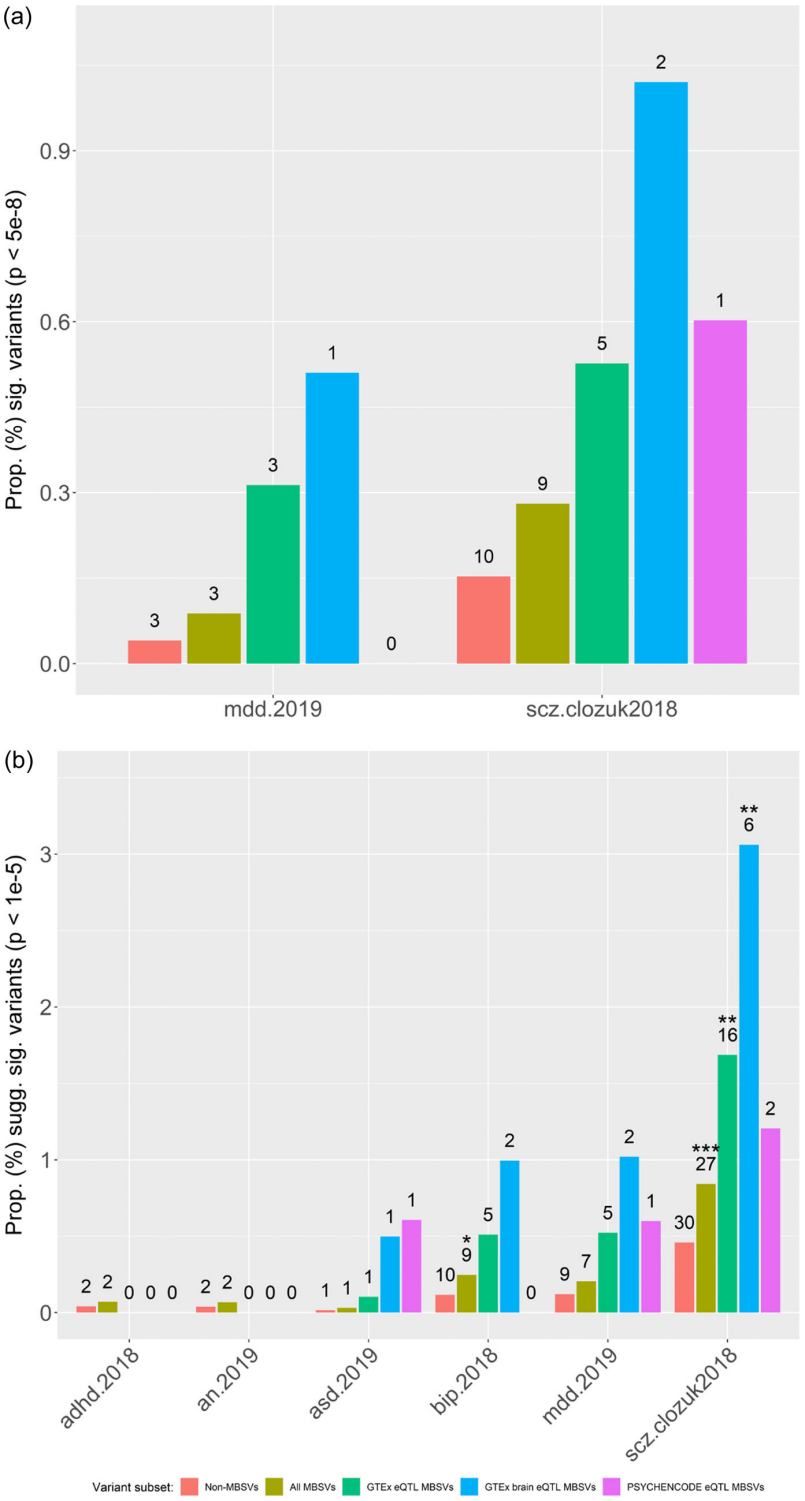
Proportions of strongly‐associated variants in each disorder. Variants were filtered for genome‐wide significance (*p* < 5 × 10^−8^) (a) or suggestive significance (*p* < 1 × 10^−5^) (b). Proportions of all strongly‐associated MBSVs (gold) and eQTL‐annotated MBSVs (GTEx in green; GTEx brain in blue; PSYCHENCODE in pink) were compared to the proportion of strongly‐associated 3′UTR‐localized non‐MBSVs (red) using Fisher's exact test. Only disorders with at least one significant MBSV are shown. Numbers represent counts of significant variants. *p < 0.05; **p < 0.01; ***p < 0.001. MBSV, miRNA binding site variant; SCZ, schizophrenia.

We next aimed to assess whether MBSVs were enriched for functional annotation with the CADD score. For every variant, we retrieved its CADD score, then compared the ECDF of all brain‐expressed 3′UTR variant CADD score ranks to the score ranks of MBSVs, GTEx eQTL MBSVs, GTEx brain eQTL MBSVs and PSYCHENCODE eQTL MBSVs. We obtained scores from both the most recent version of CADD (v1.6) as well as the original published version (v1.0). The use of both versions of the database was due to the addition of miRNA binding site scores from both TargetScan and mirSVR to the CADD v1.1 algorithm; as such, the later versions of the scores may be biased for MBSVs. K–S tests revealed a significant deviation of ECDFs for all classes of MBSVs in both versions of CADD except for GTEx brain eQTL MBSVs in the CADD v1.6 database (Supporting Information: Table 1 and Figure 1). Median regression further revealed that the median CADD score rank was significantly reduced for all classes of MBSVs in the original version of the CADD database, which held true for just PSYCHENCODE MBSVs in v1.6 (Supporting Information: Tables 4 and 5 and Figure 2).

### 3.2 MBSVs are enriched in several psychiatric disorders

**Figure 3:**
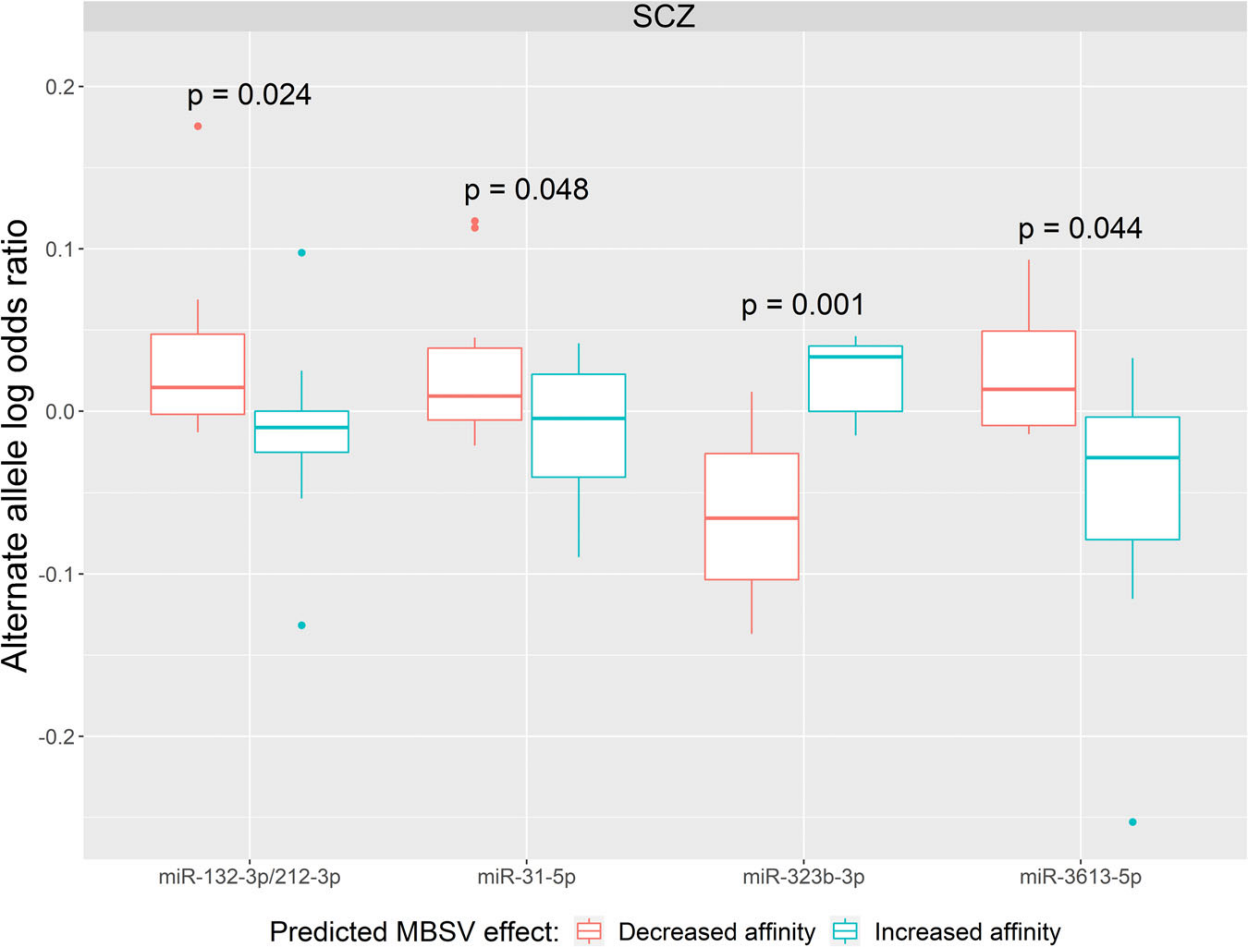
Relationship between the sign of MBSV family difference scores and SCZ effect sizes. Effect sizes were the log‐transformed odds ratios from the 2018 SCZ GWAS (Pardiñas et al., 2018), and were standardized to be relative to the alternate allele, as all difference scores were calculated for the alternate alleles. A linear regression model was fitted to determine whether the change in predicted binding site affinity attributable to the alternate allele was significantly associated with the effect size of the alternate allele. Shown are the results for the four nominally significant (*p* < 0.05) miRNA families. MBSV, miRNA binding site variant; SCZ, schizophrenia.

To determine if there was any association between MBSVs and each psychiatric disorder, we first analyzed the distribution of *p* values for all independent variants in each GWAS data set and compared these to the distributions of independent GWAS MBSVs. This revealed significantly different distributions of MBSV and non‐MBSV 3′UTR variant *p* values in SCZ, BIP, and MDD after correction for multiple tests (Figure 1 and Supporting Information: Table 2, Figure 3–10). Further to this, quantile regression revealed that for BIP, MDD, SCZ, and TS, MBSVs were associated with significantly higher negative log_10_ *p* values relative to non‐MBSV 3′UTR variants at one or more of the tested quantiles: 50th (median), 90th, 99th, and 99.9th percentiles (Supporting Information: Table 6). In support of these findings, we found that for SCZ there was a nominally significant enrichment of genome‐wide associated (*p* < 5 × 10^−8^) MBSVs (Figure 2 and Supporting Information: Table 8), with 9 out of 3207 (0.28%) MBSVs being genome‐wide significant compared to 1 out of 3326 (0.030%) of all independent non‐MBSV GWAS variants located within brain‐expressed 3′UTRs (OR = 9.33; *p* = 0.010; FDR = 0.188). While not reaching significance following correction for multiple tests, when a less rigorous variant‐level *p* value threshold was used (*p* < 1 × 10^−5^) as a measure of suggestive significance, SCZ displayed a significant enrichment of significant MBSVs (27/3207 [0.84%] vs. 3/3326 [0.090%]; OR = 9.33; *p* = 3.43 × 10^−6^; FDR = 1.24 × 10^−4^), as did BIP (9/3657 [0.25%] vs. 1/4936 ([0.020%]; OR = 12.1; *p* = 2.84 × 10^−3^; FDR = 2.56 × 10^−2^). At this liberal significance threshold, both SCZ and BIP retained significant enrichments for GTEx MBSVs, and SCZ further remained significant when testing for the enrichment of brain‐specific GTEx MBSVs (Figure 2 and Supporting Information: Table 8).

We next aggregated all MBSV *p* values for each disorder using the ACAT method. Again, there was a significant association (Bonferroni‐corrected *p* < 0.05) between MBSVs and SCZ and BIP, as well as MDD and ADHD (Supporting Information: Table 9). Furthermore, BIP, MDD, and SCZ retained significant aggregated associations with GTEx MBSVs and brain‐specific GTEx MBSVs; SCZ and MDD were also significantly associated with PSYCHENCODE MBSVs. Due to the significant known genetic correlation between SCZ, MDD, and BIP (SCZ‐BIP: 68%; SCZ‐MDD: 34%; MDD‐BIP: 35%) (Brainstorm Consortium et al., 2018), we also performed several meta‐analyses of these *p* values using Stouffer's weighted *Z*‐score method: SCZ and MDD; SCZ and BIP; SCZ, MDD, and BIP; and a final meta‐analysis across all disorders. Each of these meta‐analyses revealed a highly significant association of MBSVs, as well as all eQTL‐annotated subsets of MBSVs (Supporting Information: Table 11).

We also sought to determine if MBSVs displayed a significantly different effect size compared to all GWAS variants. We calculated the ECDF of the absolute log‐transformed effect sizes (log‐OR or *β*) for MBSVs and all GWAS variants for each disorder. We found that for all GWAS there was a significantly different distribution of effect sizes for MBSVs and all subsets of MBSVs relative to non‐MBSVs in brain‐expressed 3′UTRs (Supporting Information: Table 3 and Figures 11–19). This finding was recapitulated with the quantile regression analysis, where for every GWAS, almost all quantiles tested (median, 90th, 99th, and 99.9th percentiles) showed a significant difference between MBSVs and non‐MBSVs (Supporting Information: Table 7 and Figures 20–28). Interestingly, for all significantly different quantiles, the regression slope was negative, indicating that MBSVs tended to have smaller absolute effect sizes compared to non‐MBSVs, echoing the findings from the CADD rank score analyses.

### 3.3 Variants affecting the binding of specific miRNA families are associated with psychiatric disease

We next aggregated MBSV *p* values separately for individual miRNA families in each disorder. We identified a total of 40 unique miRNA families reaching genome‐wide significance (Bonferroni‐corrected *p* < .05) in one or more of SCZ, BIP, MDD, and ADHD (Supporting Information: Table 10). Of these, six miRNA families (miR‐335‐5p, miR‐21‐5p/590‐5p, miR‐361‐5p, miR‐577, miR‐31‐5p, and miR‐22‐3p) were highly significantly associated with both SCZ and MDD. Again, we further meta‐analyzed the results for SCZ‐MDD, SCZ‐BIP, SCZ‐MDD‐BIP, and all disorders. A total of 52 unique families were associated with one or more of these meta‐analyses (Supporting Information: Table 12). Of these, eight families, miR‐335‐5p, miR‐577, miR‐21‐5p/590‐5p, miR‐361‐5p, miR‐31‐5p, miR‐22‐3p, miR‐132‐3p/212‐3p, and miR‐194‐5p were consistently genome‐wide significant in each analysis.

### 3.4 Relationship between MBSV difference scores and GWAS effect sizes

We next investigated whether the difference scores for MBSVs in each associated miRNA family in each psychiatric disorder demonstrated a relationship with the variants’ effect sizes. We only considered miRNA families that were significantly associated in the ACAT analyses. The effect sizes were standardized such that they were all relative to the alternate allele, as the difference scores were also calculated relative to the alternate allele. A linear regression analysis revealed no significant relationship between difference scores and effect sizes for any miRNA family in any disorder, although miR‐31‐5p did display a nominally significant relationship in MDD whereby elevated binding affinity was associated with more positive effect size, that is, the increased risk (*p* = 0.021, FDR = 0.614) (Supporting Information: Table 17 and Figure 29). However, we also investigated whether the sign of the difference scores were associated with effect size. In this analysis, we identified four nominally significant (*p* < 0.05) miRNA families, miR‐323b‐3p, miR‐132‐3p/212‐3p, miR‐3613‐5p, and miR‐31‐5p in SCZ, with the miR‐323b‐3p family being the most strongly associated (*p* = 0.00149, FDR = 0.079), where a gain of binding affinity in the presence of the alternate allele was associated with greater risk (Figure 3 and Supporting Information: Table 18). The other three nominally significant miRNA families showed the inverse relationship, where elevated binding affinity was associated with reduced risk.

### 3.5 Analysis of gene sets affected by MBSVs

To identify whether MBSVs had any aggregated effects within disease‐relevant pathways, we utilized MAGMA to perform gene‐set association analyses for each GWAS, subset by MBSVs. Gene sets were all gene ontology sets obtained from the molecular signatures database (MSigDB). At an FDR threshold of 0.05, we identified 2, 2, and 1 gene sets in ASD, SCZ, and AN, respectively, that were significantly enriched for association with MBSVs or eQTL MBSVs. These included “mRNA binding” and “retromer complex” in SCZ for GTEx brain eQTL MBSVs and all GTEx MBSVs, respectively; “protein phosphatase binding” and “nuclear periphery” in ASD for PSYCHENCODE MBSVs; and “carbon nitrogen ligase activity with glutamine as amido‐N‐donor” in AN for all MBSVs (Supporting Information: Table 13). At the gene level, 39 unique genes affected by MBSVs and eQTL MBSVs were significantly associated with one or more of ADHD, AN, BIP, MDD, and SCZ (Bonferroni‐corrected *p* <  0.05) (Supporting Information: Table 15). The most significant of these findings were in SCZ, with several genes, such as *ZKSCAN8*, *CNNM2*, and *SUFU*, and *DGKI* being affected by multiple independent MBSVs.

**Figure 4:**
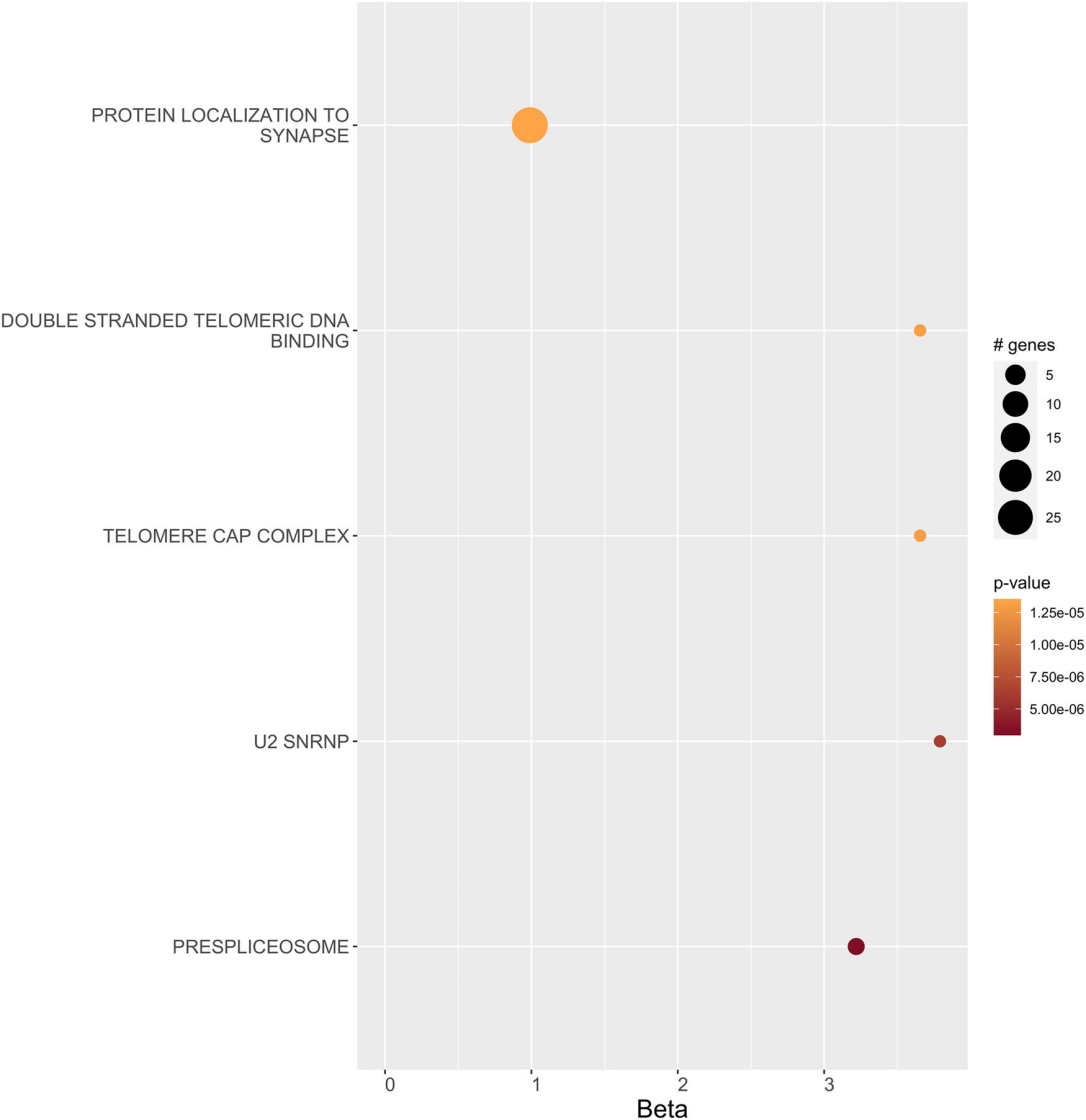
Gene ontology sets that were associated (FDR < 0.1) with all MBSVs in a gene‐level meta‐analysis of SCZ and BIP conducted in MAGMA. BIP, bipolar disorder; FDR, false discovery rate; MBSV, miRNA binding site variant; SCZ, schizophrenia.

Again, we investigated whether any gene ontology sets displayed joint association when the gene‐level results from MAGMA for these disorders were meta‐analyzed. We conducted four different meta‐analyses: SCZ and BIP; SCZ and MDD; SCZ, MDD, and BIP; and all disorders (Figure 4 and Supporting Information: Tables 14 and 16). SCZ‐BIP was strongly associated with “prespliceosome” (all MBSVs), “retromer complex” (GTEx MBSVs), and “mRNA binding” (GTEx brain eQTL MBSVs). The all disorder analysis found associations with “protein acetylation” (PSYCHENCODE MBSVs) and “regulation of receptor biosynthetic process” (all MBSVs). At a slightly more liberal FDR threshold, several more gene sets were significant, most notably “protein localization to synapse” in SCZ‐BIP for all MBSVs, with 27 affected genes (*p* = 1.35 × 10^−5^, FDR = 0.0574), and “regulation of RAS protein signal transduction” in the all disorder analysis for all MBSVs, with 73 genes affected (*p* = 1.12 × 10^−5^, FDR = 0.0574).

## 4 DISCUSSION

In this study, we aimed to investigate whether common genetic variation within miRNA binding sites contributes toward the pathophysiology of psychiatric disorders. Psychiatric disorders are typically complex, polygenic conditions with many small common variants, each with relatively small effect sizes, contributing toward overall risk. The majority of risk variants tend to lie outside coding regions and are thought to instead affect gene regulatory sequences, such as promoters and enhancer regions (Bray & O'Donovan, 2019). Concordant with this hypothesis, miRNA are increasingly being studied for their roles in psychiatric disease, as biomarkers, and as potential novel treatment targets (Beveridge & Cairns, 2012; M. Geaghan & Cairns, 2015; B. H. Miller & Wahlestedt, 2010). Both genetic and experimental biological studies have demonstrated a significant role for miRNAs in brain development, neuronal function, and the pathophysiology of psychiatric disease. For example, the genetic locus containing the miR‐137 host gene *MIR137HG* is host to one of the most significant common genetic associations with SCZ (Pardiñas et al., 2018; Schizophrenia Working Group of the Psychiatric Genomics Consortium, 2014). Further biological evidence suggests that this miRNA is responsible for regulating the formation and maturation of synapses, and its overexpression has been observed to result in reduced synapse formation and synaptic transmission (He et al., 2018). It has also been implicated in other psychiatric and neurological conditions, including ASD, Huntington's disease, and Rett syndrome (Mahmoudi & Cairns, 2017). Other miRNAs, as well as their biogenesis machinery have frequently been implicated in SCZ and other psychiatric disorders, as well as in the regulation of synaptic and neuronal function, such as the miR‐132/212 cluster and miR‐185 (Beveridge et al., 2010; Earls et al., 2012; M. Geaghan & Cairns, 2015; M. P. Geaghan et al., 2019; B. H. Miller et al., 2012). We hypothesized that miRNA binding site sequences, present within 3′UTRs of protein‐coding genes, may represent yet another class of regulatory sequences that are affected by common variants that predispose toward psychiatric disease. While previous studies have investigated the role of these variants within SCZ (Devanna et al., 2018; Hauberg, Holm‐Nielsen, et al., 2016; Hauberg, Roussos, et al., 2016) a large study of MBSVs across several psychiatric disorders has not been performed to date. To this end, we identified variants predicted to affect miRNA binding sites. As miRNA prediction algorithms suffer from relatively high false‐positive rates (Lewis et al., 2005), we only considered binding sites predicted by TargetScan and at least one of the other two algorithms employed by dbMTS (RNAhybrid and miRanda). We constructed a difference score based on the TargetScan scores by subtracting the best (most negative) score for the reference allele from the best score for the alternate allele. This differed from the precalculated difference score supplied in the dbMTS database. We reasoned that the most conservative score would reflect the difference between the miRNA–mRNA interactions with the highest affinities for each allele, in contrast to the dbMTS approach of using the maximum absolute difference in scores. We further filtered out variants with small difference scores (absolute score < 0.2). The remaining 8334 variants were considered “true” MBSVs.

We found evidence for significant enrichment of MBSV association within multiple psychiatric disorders, with the most consistent findings being for SCZ, BIP, and MDD. This suggests that MBSVs are a significant source of genetic risk in both these disorders, and are represented more frequently than would be expected by chance, even among other 3′UTR variants. We further dissected our data by investigating only MBSVs annotated as eQTLs and specifically brain eQTLs for the gene in which they are present. While there are significantly fewer MBSVs that are also eQTLs and brain eQTLs, we still observed a significant enrichment of association of these variants compared to all non‐MBSVs for SCZ and BIP and found a significant enrichment of suggestive significant (*p* < 1 × 10^−5^) MBSVs in both GWAS as well. It should also be noted that several of the GWA studies used were supported by smaller sample sizes compared to SCZ (Grove et al., 2019; Howard et al., 2019; Pardiñas et al., 2018; Stahl et al., 2019; Watson et al., 2019), and as such, analysis of future GWAS may demonstrate a more significant enrichment of genome‐wide associated MBSVs. Another unexpected result was that MBSVs that were significant eQTLs in PSYCHENCODE were not as significantly enriched for genome‐wide‐significant variants as compared to GTEx brain eQTL MBSVs. This may in part be due to the wider range of brain tissues represented in the GTEx data set. A deeper analysis of tissue‐specific MBSVs might help to resolve this discrepancy, although such an analysis would require both higher‐powered GWAS data to identify more genome‐wide‐significant MBSVs, and the development of mature tissue‐specific miRNA target prediction algorithms and databases.

An interesting finding was that MBSVs typically display smaller effect sizes than other variants. This was partially reflected in the fact that the median CADD score ranks for MBSVs and each eQTL subcategory in v1.0 of the database were significantly lower than other non‐MBSV brain‐expressed 3′UTR variants. These results suggest that while MBSVs may be a significant contributor toward genetic risk, they typically have smaller effect sizes than other variants within 3′UTR regions. This is not completely surprising, since miRNAs are typically subtle regulators of gene expression; their role is typically framed as a “fine‐tuner” of protein expression within the cell (Selbach et al., 2008). Furthermore, a typical mRNA will possess several miRNA binding sequences within its 3′UTR; thus, a single dysfunctional miRNA may be compensated for by other miRNAs, reducing the effect size of any one MBSV. Environmental factors, such as cellular and physical stress, can also influence miRNA expression (Lukiw & Pogue, 2007; Mannironi et al., 2013; Rocha, 2007), and thus there may be significant genotype‐by‐environment (GxE) interactions present, whereby MBSVs are only pathologically relevant within the context of other environmental risk factors for psychiatric disease. A significant caveat with the CADD scores was that they are calculated based on many functional annotations of known SNVs, and from v1.1 onward these annotations include the TargetScan and mirSVR (Betel et al., 2010) target prediction algorithms. Thus, these scores are likely biased towards MBSVs, hence our inclusion of the v1.0 CADD database within our analyses.

By aggregating *p* values assigned to each MBSV for each disorder, we identified a highly significant association between this class of variants and SCZ, BIP, MDD, and ADHD. Several specific miRNA families also showed an association with each of these disorders. Perhaps the most interesting finding was a strong association between MBSVs affecting the miR‐132‐3p/212‐3p family and SCZ; the expression of this miRNA family has previously been associated with SCZ, and it is known to play a significant role in neuronal function. Specifically, miR‐132‐3p/212‐3p targets are enriched for pathways associated with synaptic plasticity and neuronal migration pathways such as Reelin signaling (B. H. Miller et al., 2012). Furthermore, miR‐132‐3p expression increases during early post‐natal development in mice and is positively regulated by N‐methyl‐D‐aspartate receptor activation (B. H. Miller et al., 2012). In the current study, this miRNA family retained this association within each of the four cross‐disorder meta‐analyses we performed. This result represents a significant finding that strengthens the support for the role of the miR‐132/212 cluster in the pathophysiology of SCZ.

Interestingly, little evidence for association was observed for MBSVs affecting the miR‐137 family in any disorder. This miRNA has been of particular interest in the context of SCZ, primarily due to the highly significant genetic association within its genetic locus (Pardiñas et al., 2018; Schizophrenia Working Group of the Psychiatric Genomics Consortium, 2014). This does not suggest that this miRNA has been erroneously associated with SCZ; rather, it only suggests that genetic variants affecting miR‐137 binding do not significantly affect SCZ risk. As discussed above, various factors, including compensation by other miRNA binding sites, and GxE interactions, may mitigate the effects of individual variants on the binding of any single miRNA. Instead, variants affecting miR‐137 expression and the expression of its target genes may be more important for determining disease risk. Indeed, miR‐137 targets (as predicted by TargetScan v5) were enriched for genome‐wide significant associations in the 2014 PGC SCZ GWAS (Schizophrenia Working Group of the Psychiatric Genomics Consortium, 2014). Furthermore, a variable‐number tandem repeat (VNTR) present in the *MIR137HG* locus has been demonstrated to regulate miR‐137 expression, with shorter repeats linked to higher miR‐137 expression and increased SCZ risk (Pacheco et al., 2019). However, it should also be emphasized that this locus also contains the *MIR2682* miRNA gene, the *MIR137HG* long noncoding RNA that hosts these two miRNAs, and *DPYD*. Interestingly, *DPYD* (dihydropyrimidine dehydrogenase) has previously been linked to autism (Ben‐David et al., 2011; Carter et al., 2011). As such, it is also possible that miR‐137 is not the major causal factor influencing risk for SCZ.

Interestingly, the miRNA family miR‐335‐5p was the top‐most associated family when aggregating MBSV *p* values within both SCZ and MDD. This miRNA has previously been associated with depression, whereby it was found to be downregulated in PBMCs in individuals with MDD, and was further found to regulate the depression‐associated glutamate metabotropic receptor 4 (*GRM4*) and was upregulated in response to treatment with the antipsychotic citalopram (J. Li et al., 2015). This miRNA was also among those upregulated in a previous study from our laboratory (Beveridge et al., 2010). Thus, this miRNA may be particularly important for these conditions and represents an interesting focus for future research into miRNAs in psychiatric disorders and their treatment.

We also found suggestive evidence for a relationship between MBSVs affecting miR‐323b‐3p binding and SCZ risk. The negative association between miR‐323b‐3p difference score and SCZ effect size suggests that increased binding affinity for this miRNA may be associated with elevated risk for SCZ. This miRNA has not been thoroughly studied in the context of psychiatric disease; the current literature does suggest it has a significant role in promoting neuronal apoptosis in the context of ischemia by targeting *BRI3* and *SMAD3* mRNA (Che et al., 2018; Yang et al., 2015). As such, it is possible that this miRNA is involved in regulating the proliferation of cells in the developing brain. Importantly, mature miRNAs in this family are enriched in the rat brain (Kim et al., 2004), and are located within a maternally imprinted cluster of miRNAs in the DLK1‐DIO3 at 14q32 locus that were downregulated in SCZ and a two‐hit rat model of maternal immune activation and adolescent cannabis exposure (Gardiner et al., 2012; Hollins et al., 2014). Furthermore, this miRNA family has been shown to regulate *FMR1* gene expression—a gene vital for dendritic spine and synapse development, and loss of function of which results in the debilitating neuropsychiatric disorder Fragile X syndrome (Nimchinsky et al., 2001; Yi et al., 2010). Together, this evidence suggests that this miRNA is important in neuronal development and function, and its role in SCZ is worthy of further study.

At the gene set level, we found suggestive evidence implicating pathways relating to neuronal function and nervous system development which were associated with SCZ and BIP. Meta‐analysis of SCZ and BIP identified suggestive associations (FDR < 0.1) of “protein localization to synapse” and “negative regulation of synapse development” being observed among MBSVs and eQTL MBSVs, respectively. These results further support our hypothesis of the involvement of MBSVs in psychiatric disease and neuronal function and suggest that this class of variants may have a significant influence over the expression of genes involved in synaptic function. This fits with previous research suggesting that several psychiatric disease‐associated miRNAs are potent regulators of synaptic activity and neuronal function. For example, the mature miRNAs arising from the miR‐132/212 cluster have been demonstrated to influence basal synaptic transmission and aspects of long‐term potentiation (LTP) in the HIP and neocortex in mice (Remenyi et al., 2013), and miR‐132 specifically has been shown to regulate dendritic complexity and spine density, with subsequent effects on GABAergic and glutamatergic activity in olfactory bulb neurons (Pathania et al., 2012). Similarly, miR‐137 has been shown to affect LTP, the synaptic vesicle pool, and aspects of learning and memory in mice (Siegert et al., 2015). It has also been shown that certain miRNAs, such as miR‐134, can themselves be localized to the synapse (Schratt et al., 2006), and their biogenesis and silencing capacities can even be regulated by synaptic activity (Ashraf et al., 2006), thus allowing for fine control over protein synthesis at the synapse in response to neuronal stimuli. Our results add a new level of complexity to this regulatory system, suggesting that genetic variants affecting miRNA function may also influence processes of synaptic protein expression in psychiatric disorders.

We also repeated these analyses with nonpsychiatric human traits, including type 2 diabetes, BMI, height, and CAD. In most cases, we found a similar trend with a general enrichment of association between MBSVs and each trait and significant associations between specific families of MBSVs and each trait. Interestingly, we also observed significant associations between each trait and relevant MBSV‐affected gene sets; for example, “lipid localization” was significantly associated with blood eQTL MBSVs in BMI. These results suggest that the importance of MBSVs to the genetics of human traits is not specific to psychiatric disorders but may be a more general phenomenon. This is not entirely unexpected given that miRNAs have been shown to be functionally significant in a plethora of human traits, conditions, and diseases, such as various types of cancer, neurological diseases such as Alzheimer's and Huntington's disease, and each of the nonpsychiatric traits we have studied here (M. Geaghan & Cairns, 2015; Johnson et al., 2008; Lee et al., 2011; Swarbrick et al., 2019; Tong & Nemunaitis, 2008; W.‐X. Wang et al., 2011). Thus, the methods employed in this study can be used to investigate the roles of MBSVs (and other noncoding regulatory regions) in a wide range of human traits, and could be useful in developing novel hypotheses and treatments for human diseases beyond psychiatric disorders.

Together, these results represent promising support for the role of MBSVs in regulating neuronal function in psychiatric disorders. However, the relatively small number of MBSVs compared to all common variants typically assessed in GWAS, and the difficulty of confidently predicting true miRNA binding sites means that the power to detect the effects of these variants is relatively low. These two factors represent the greatest limitations to this study and will be the most important areas for improvement in future work. Improving the power to detect the genetic contribution of MBSVs to human traits and disorders relies on the continued escalation of GWAS cohort sizes. Thus, repeating these analyses upon release of larger GWAS summary statistics, as well as in GWAS of different ethnic backgrounds, and upon the development of improved miRNA target prediction algorithms will be vital to the further exploration of the roles of MBSVs in complex diseases. The other significant limiting factor—the relatively high false positive rate of current miRNA target site prediction algorithms (Lewis et al., 2005)—remains a continuing issue in miRNA biology research, and while we have attempted to mitigate this through the use of data from multiple prediction algorithms, caution must be used when interpreting these results. These issues arise partly from the short seed region that is most important in animal miRNA target recognition, and the variable efficacy of miRNA binding between target sites and across tissues. Importantly, while we attempted to investigate the effects of tissue‐specific variants affecting miRNA binding sites, the lack of tissue‐specific miRNA binding site prediction limits how accurate this approach can be. As future experimental and computational datasets become available, it may be possible to improve the accuracy of miRNA binding site prediction in a tissue‐specific manner, and thus improve the ability to detect MBSVs important for disease.

Despite these limitations, we believe our approach provides a useful platform from which future studies can be performed. Importantly, our approach can be applied to other complex disorders for which GWAS summary data are available. Furthermore, while this study focussed on common variation, we suspect that rare MBSVs with greater effect sizes exist and that future studies focussing on these may shed more light on the influence of this class of regulatory variants in complex diseases. Nevertheless, these results suggest that variants that influence the binding of miRNAs to the 3′UTRs of mRNAs are a significant source of genetic risk for complex disorders, and in the case of psychiatric disorders they may be affecting normal neuronal function by disrupting the localization of proteins to the synapse. Further study of these variants may help to elucidate the complex molecular landscape of psychiatric and other complex disorders that ultimately may drive the development of novel treatments.

## CONFLICT OF INTEREST

The authors declare no conflict of interest.

## Supporting information

Supporting information.

Supporting information.
