# Supplementary material for: MicroRNA binding site variation is enriched in psychiatric disorders
Source: Hum Mutat. 2022 Oct 23;43(12):2153–69. doi: 10.1002/humu.24481 (PMC10947041; doi:10.1002/humu.24481)
Supplement: Supplementary file 2 — Supporting information. [file HUMU-43-2153-s001.pdf]

MicroRNA binding site variation is enriched in psychiatric disorders

Supplementary Information

Michael P. Geaghan (PhD)[1], *William R. Reay (B. Biomed. Sci. (Hons))*[1,2], *Murray J. Cairns (PhD)*[1,2]

[1] The University of Newcastle, Callaghan, New South Wales, Australia

[2] Centre for Brain and Mental Health Research, Hunter Medical Research Institute, New Lambton, New South Wales, Australia

Analysis of non-psychiatric traits

For comparison and to determine whether these phenomena were characteristic of psychiatric disorders in particular, or of complex human traits in general, we performed the same analyses on several non-psychiatric traits. We identified similar trends within GWAS of height, CAD, BMI, and T2D, including enrichment of strongly-associated MBSVs, smaller effect sizes of MBSVs compared to non-MBSV 3'UTR variants, as well as aggregation of association within specific miRNA families and relevant gene sets, such as blood eQTL MBSVs in BMI being associated with the gene ontology set "*lipid localisation*". For further details, see the supplementary text.

Proportions of MBSVs in non-psychiatric trait GWAS

Similar to the psychiatric disorder GWAS, we found that MBSVs accounted for approximately 0.068% of all GWAS variants (SD = 0.0081%), with between 1,770 and 9,597 independent MBSVs out of 95,760 – 803,539 total independent variants (mean proportion of independent MBSVs = 0.77%, SD = 0.46%).

Differential distribution of CADD score ranks

CADD scores were found to be significantly differentially distributed for all classes of MBSVs in each version of the CADD database, except for pancreas eQTL MBSVs in type 2 diabetes (Supplementary Tables 19a-b). Interestingly, a significant effect on the median CADD score rank was only observed in v1.6 of the CADD database; except for the all blood-expressed MBSV class, which was non-significant, all other MBSV classes, both blood and pancreas-expressed, were associated with smaller CADD score ranks, mirroring the effect observed in psychiatric disorders (Supplementary Tables 22a-b and 23a-b).

Enrichment of MBSVs in each non-psychiatric trait GWAS

We found significantly altered p-value distributions for all MBSV classes in all non-psychiatric GWAS except for height (Supplementary Tables 20a-b). Quantile regression revealed that for each non-psychiatric trait, at least one class of MBSVs were associated with elevated negative log_{10} p-values at multiple tested quantiles (Supplementary Tables 24a-b). Absolute log-effect sizes were also significantly differentially distributed for most comparisons, except for: all MBSVs in BMI; GTEx eQTL MBSVs in height; and GTEx blood-specific eQTL MBSVs in BMI and height (Supplementary Tables 21a-b). Quantile regression showed that for BMI, CAD, and T2D (BMI adjusted and unadjusted, both relative to blood and pancreas gene and miRNA expression), multiple MBSV classes were associated with smaller absolute log-effect sizes at multiple quantiles; for height, only GTEx blood eQTL MBSVs were associated with reduced effect sizes at the 99.9th percentile (Supplementary Tables 25a-b).

Similar to the psychiatric analyses, no significant enrichments of genome-wide significant variants was observed in any non-psychiatric trait. However, at the liberal GWAS p-value threshold of $1x10^{-5}$, T2D (BMI adjusted and unadjusted, both relative to blood and pancreas gene and miRNA expression), all MBSVs and GTEx eQTL MBSVs were significantly enriched; unadjusted T2D, relative to both blood and pancreas expression also showed an enrichment of suggestive-significant GTEx blood and pancreas eQTL MBSVs, respectively. Height also showed a significant enrichment of MBSVs at a p-value threshold of $1x10^{-5}$ (Supplementary Tables 26a-b, Supplementary Figures 30-31).

When aggregating p-values of MBSVs for each trait, we found a significant association between all MBSVs and all traits except CAD; similarly, we found a significant association between all eQTL MBSVs and all traits (Supplementary Tables 27a-b). We further found that, relative to blood gene and miRNA expression, all non-psychiatric traits showed an association with GTEx eQTL MBSVs, and BMI (unadj.), CAD, and height also showed an association with GTEx blood eQTL

MBSVs. Relative to pancreas expression, T2D (BMI adj.) showed an association with GTEx pancreas eQTL MBSVs (Supplementary Table 27b).

MBSV p-value aggregation within miRNA families

Aggregation of all MBSV p-values into individual miRNA families revealed that there were 114 unique miRNA families associated with one or more of the non-psychiatric traits with respect to blood gene and miRNA expression (Supplementary Table 28a), and 30 associated with T2D (BMI adj. and unadj.) with respect to pancreas expression (Supplementary Table 28b). Height displayed the strongest associations, with miR-144-5p, miR-576-5p, miR-103-3p/107 and miR-423-5p being the most significantly associated miRNA families. Similarly, GTEx eQTL MBSVs affecting 99 unique miRNA families in blood-expressed genes were significantly associated with one or more non-psychiatric traits, and GTEx eQTL MBSVs affecting 34 unique miRNA families in pancreas-expressed genes were significantly associated with T2D. Finally, 48 families affected by blood-specific eQTL MBSVs were associated with non-psychiatric traits, and 10 families affected by pancreas-specific eQTL MBSVs were associated with T2D, respectively. Again, height demonstrated the most significant associations.

Associations between difference scores and effect sizes

We identified a single miRNA family, miR-1343-3p, that reached significance in a linear regression analysis between the difference score and the effect size of the alternate allele for height ($p = 1.71 \times 10^{-4}$, FDR = 0.0386, Bonf. p = 0.0386) (Supplementary Tables 31a and 32a). In this case, smaller difference scores, i.e. alternate alleles predicted to increase binding affinity were associated with increased height. In T2D (BMI adj., relative to pancreas expression) one miRNA family, miR-199-5p, was nominally significant, but did not pass multiple test correction (p = 0.0089, FDR = 0.55, Bonf. p = 0.58) (Supplementary Tables 31b and 32b).

Gene set association analyses

For non-psychiatric traits, we found that each trait had multiple significantly associated gene sets. For T2D (both BMI adj. and unadj., blood MBSVs) "*Semi lunar valve development*" and "*cardiac septum morphogenesis*" were significantly associated (FDR < 0.05) with eQTL MBSVs (Supplementary Table 29a); further for both, "*regulation of osteoclast development*" was associated with all MBSVs. For T2D (both MBI adj. and unadj.) MBSVs relative to pancreas-expressed miRNA and gene expression, a similar theme of heart-related gene sets was observed, including "*Semi lunar valve development*" for eQTL MBSVs, and "*cardiac chamber morphogenesis*" and "*heat morphogenesis*" for pancreas eQTL MBSVs (Supplementary Table 29b). Blood eQTL MBSVs in BMI were associated with "*lipid localisation*", while all MBSVs were associated with "*methylation dependent chromatin silencing*", "*negative regulation of protein impot into nucleus*", "*negative regulation of protein localisation to nucleus*", "*negative regulation of nucleocytoplasmic transport*", and "*acid phosphatase activity*". In CAD, all MBSVs were associated with "*cell morphogenesis involved in differentiation*", and importantly, 126 genes in this set were affected by MBSVs. In height, all MBSVs were associated with several gene sets, including "*response to vitamin A*" and "*glucose metabolic process*".

At the gene level, we observed 515 unique MBSV-affected genes associated with one or more non-psychiatric traits relative to blood expression, and 31 unique MBSV-affected genes associated with T2D (BMI adj. and unadj.) relative to pancreas expression (Supplementary Table 30a). Height again showed the most significant associations, with *VAPA*, *TNFSF14*, and *PLVAP* being among the most significant genes ($p = 1x10^{-50}$, Bonf. $p = 2.12x10^{-46}$ for all three genes). Among the genes associated with T2D (BMI adj. and unadj., pancreas gene expression), *NOTCH2* was the most significantly associated gene with both eQTL and pancreas eQTL MBSVs (Supplementary Table 30b).

Supplementary Figures

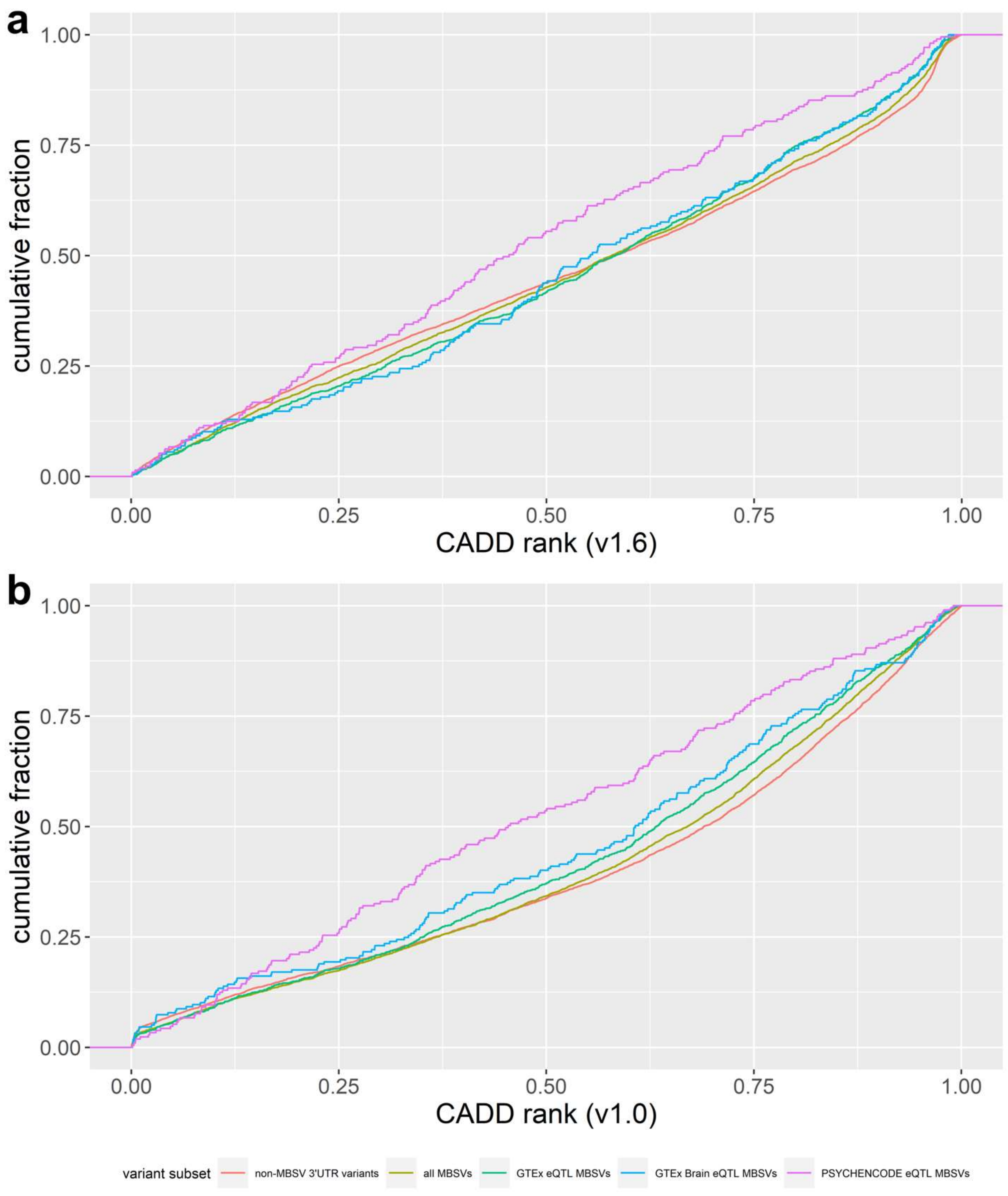

Supplementary Figure 1 | Distribution of CADD score ranks. (a, b) CADD score ranks from the latest version of the database (v1.6) (a) and the original database version (v1.0) (b) were retrieved for all MBSVs, eQTL MBSVs, and non-MBSVs within brain-expressed 3'UTR regions.

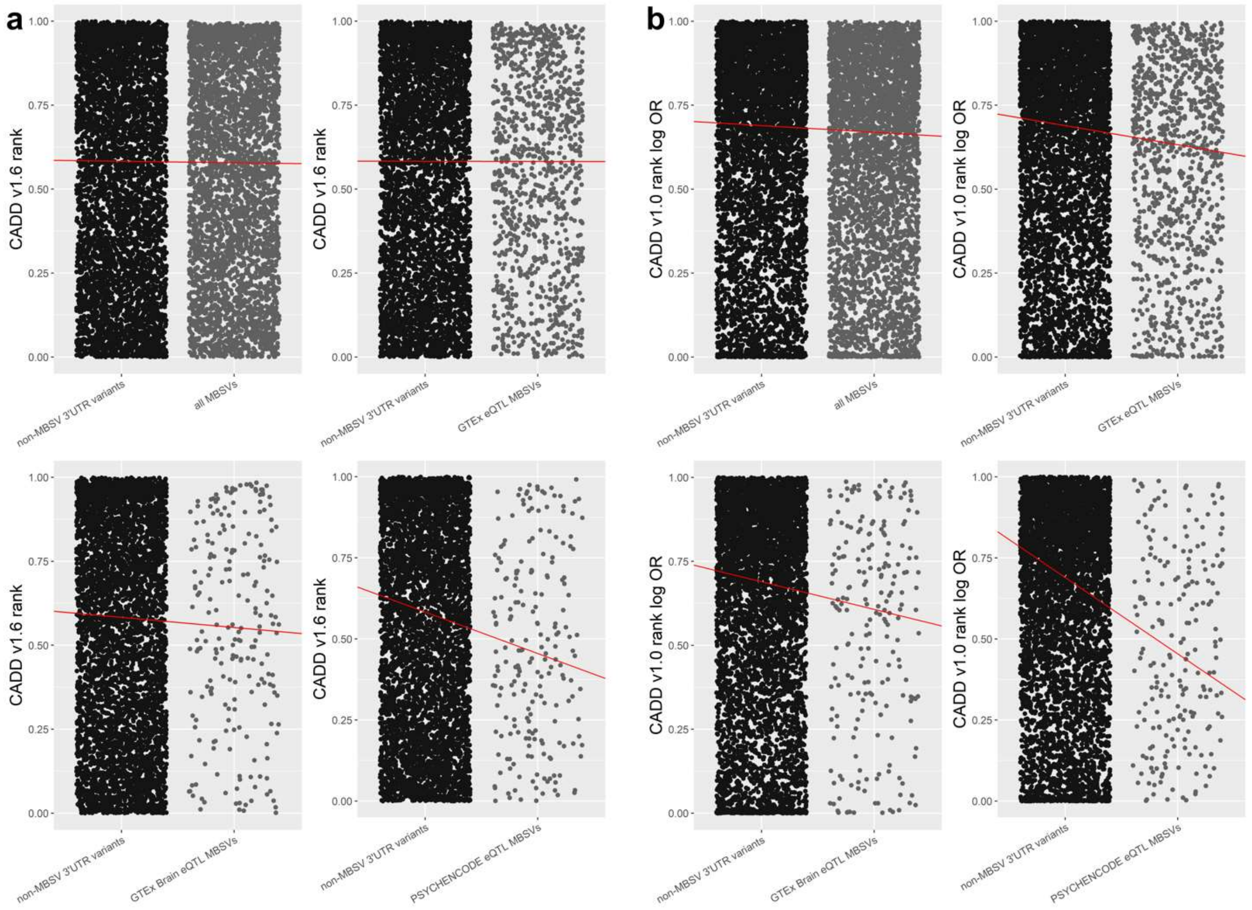

Supplementary Figure 2 | Quantile regressions of CADD score ranks from the latest version of the database (v1.6) (a) and the original database version (v1.0) (b). Median CADD score ranks were compared between non-MBSV variants within brain-expresssed 3’UTR regions and either all MBSVs, GTEx eQTL MBSVs, GTEx brain eQTL MBSVs, or PSYCHENCODE eQTL MBSVs.

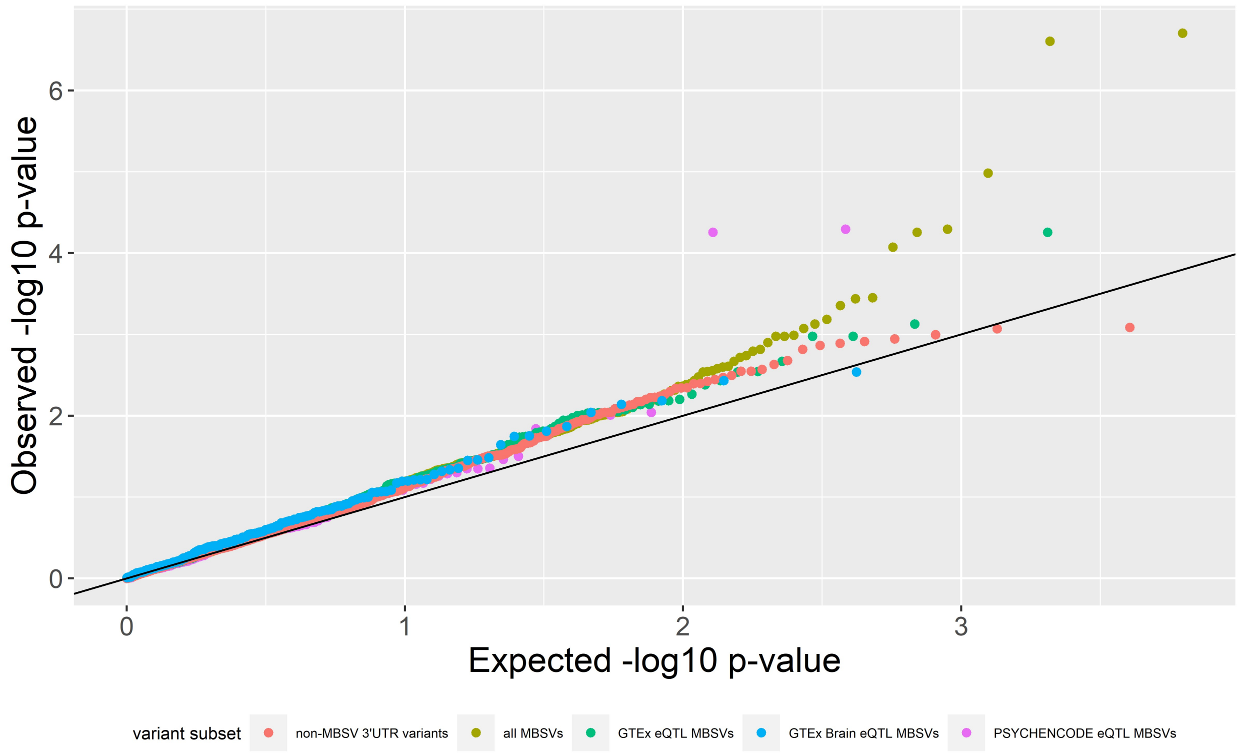

Supplementary Figure 3 | Quantile-quantile plot of -$\log_{10}$ p-values for ADHD, comparing each class of MBSVs with non-MBSV brain-expressed 3'UTR variants.

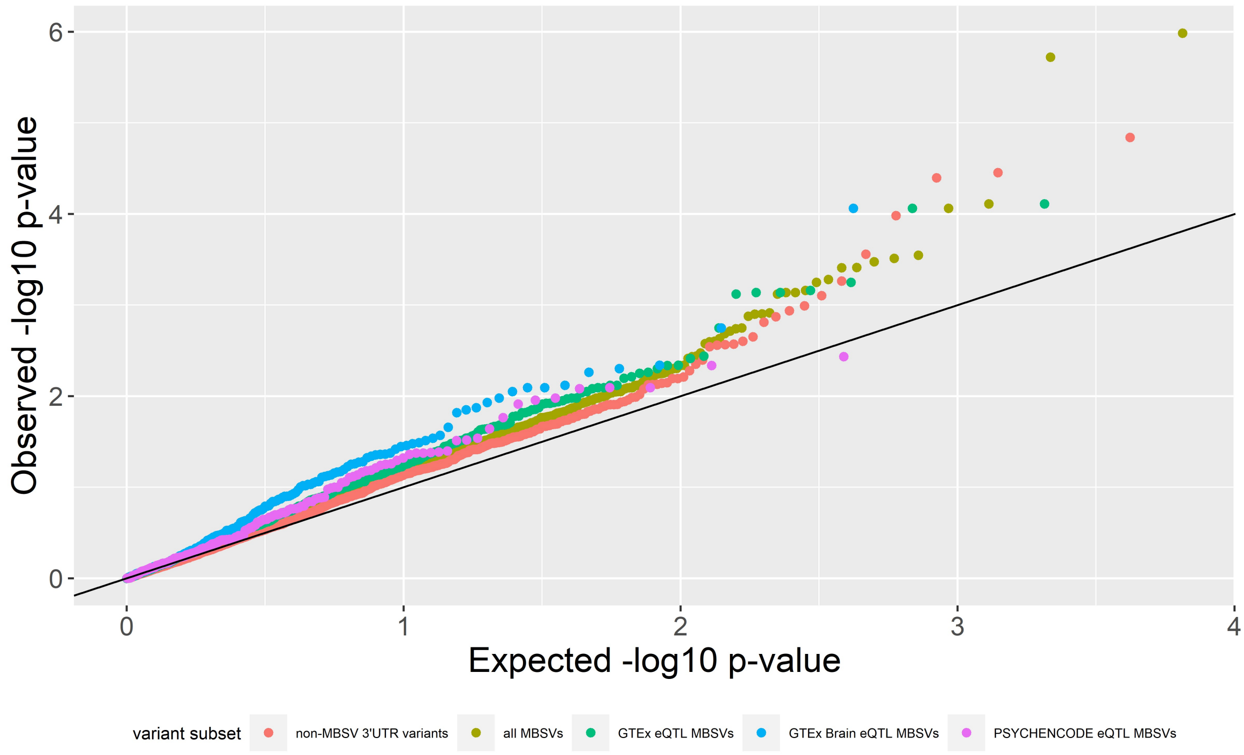

Supplementary Figure 4 | Quantile-quantile plot of -$\log_{10}$ p-values for AN, comparing each class of MBSVs with non-MBSV brain-expressed 3'UTR variants.

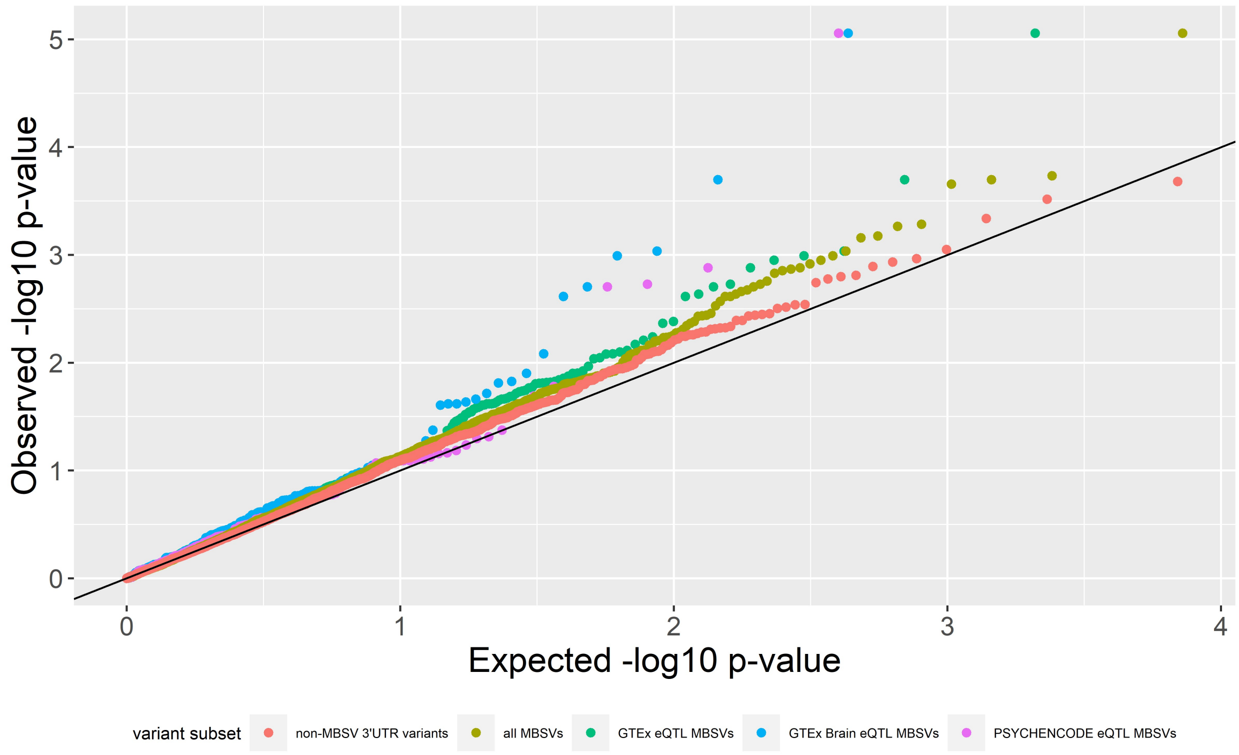

Supplementary Figure 5 | Quantile-quantile plot of -$\log_{10}$ p-values for ASD, comparing each class of MBSVs with non-MBSV brain-expressed 3'UTR variants.

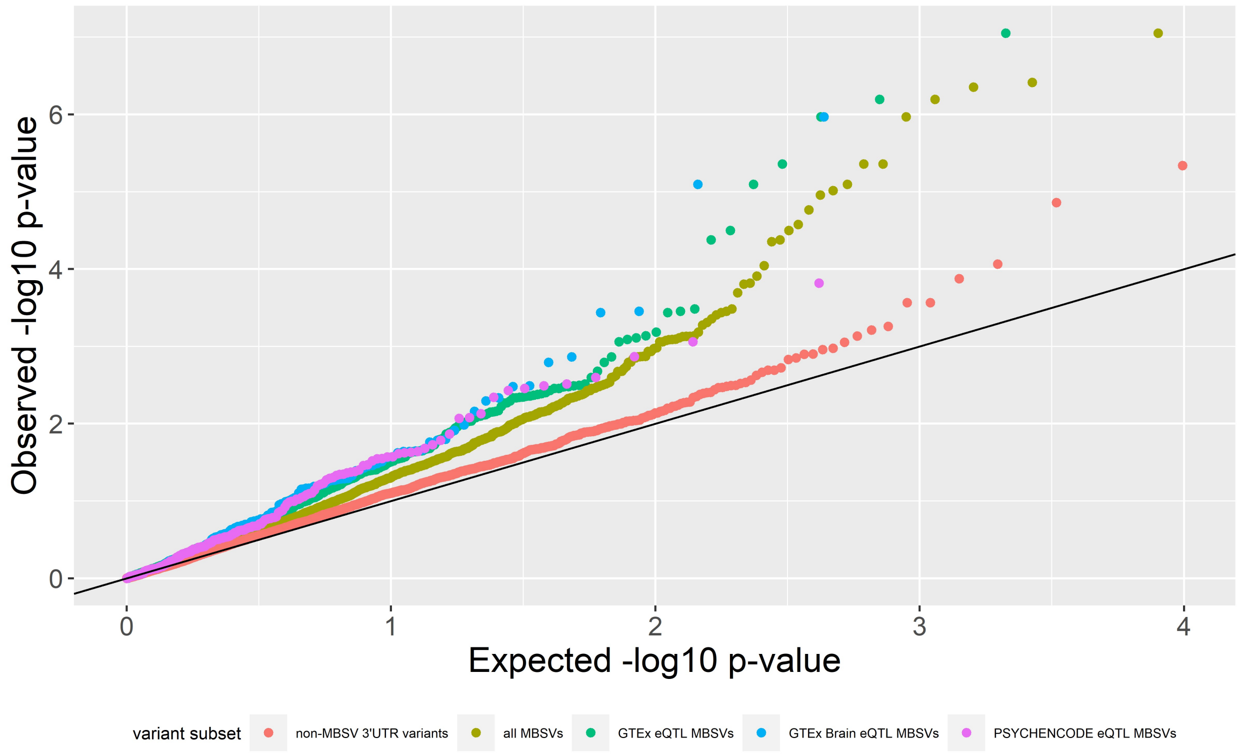

Supplementary Figure 6 | Quantile-quantile plot of -log$_{10}$ p-values for BIP, comparing each class of MBSVs with non-MBSV brain-expressed 3’UTR variants.

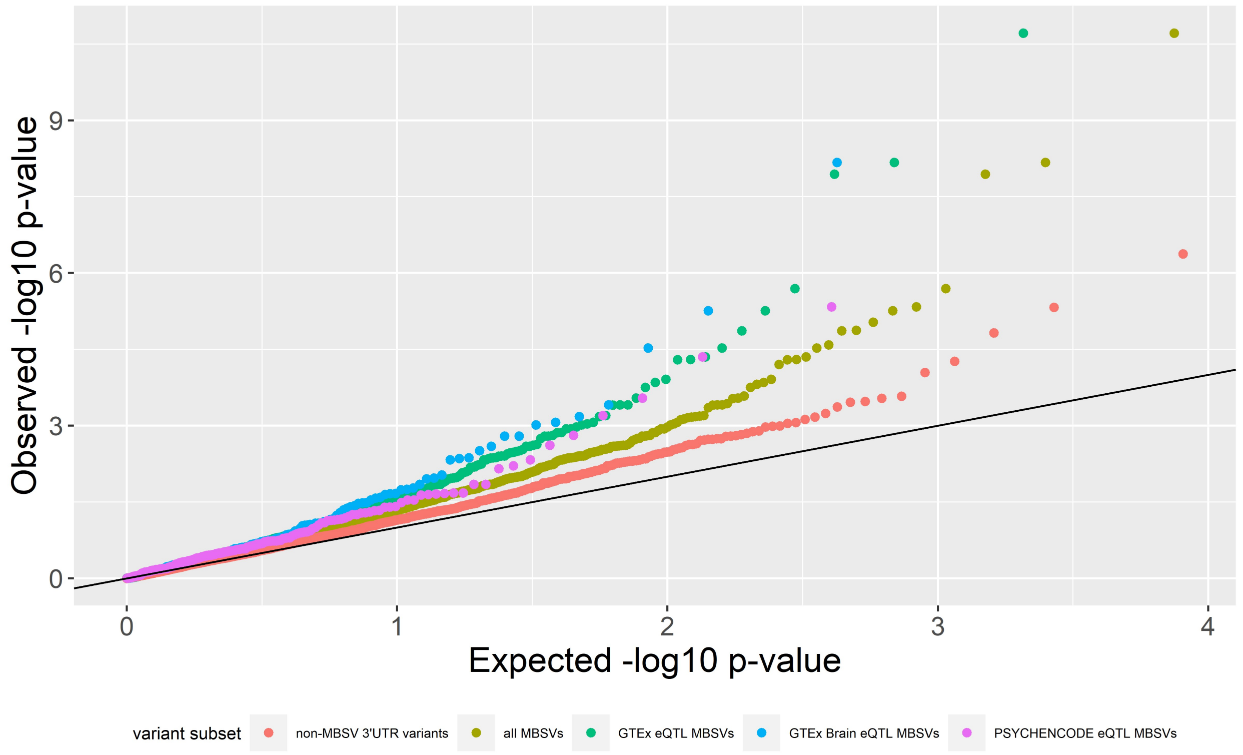

Supplementary Figure 7 | Quantile-quantile plot of -$\log_{10}$ p-values for MDD, comparing each class of MBSVs with non-MBSV brain-expressed 3’UTR variants.

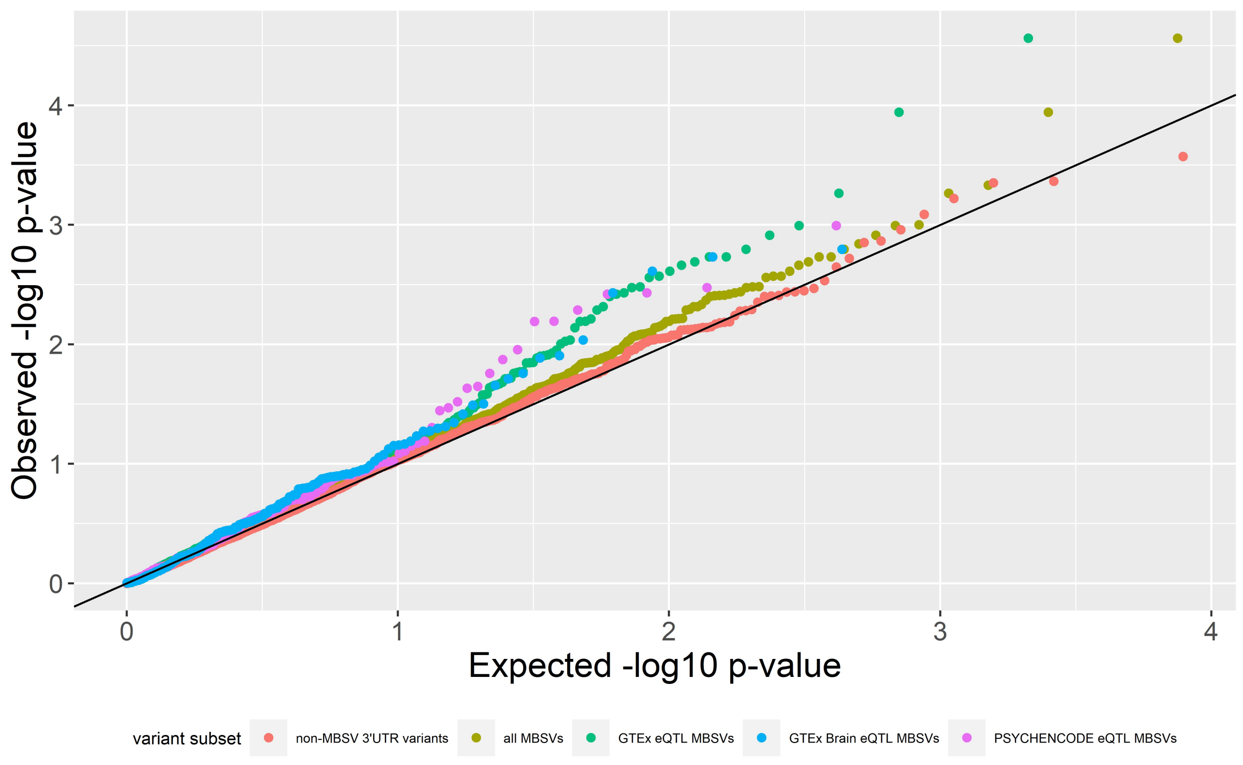

Supplementary Figure 8 | Quantile-quantile plot of -$\log_{10}$ p-values for OCD, comparing each class of MBSVs with non-MBSV brain-expressed 3'UTR variants.

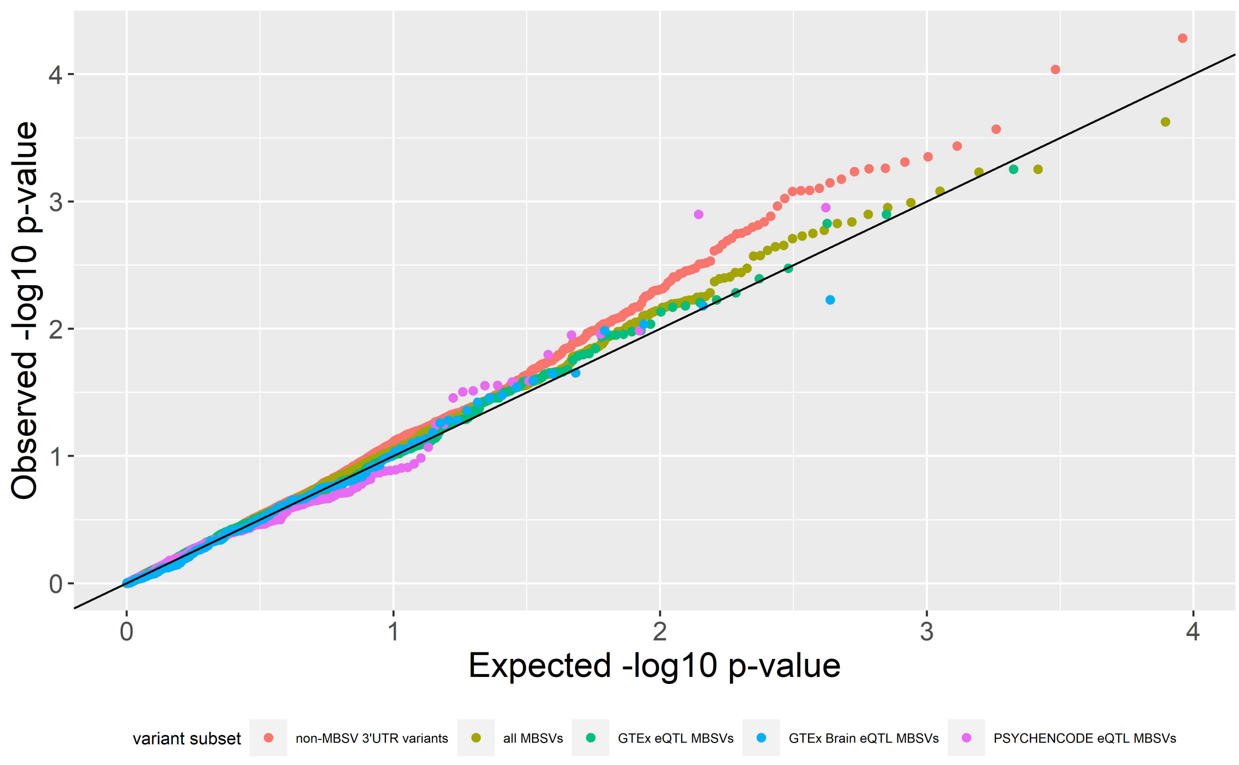

Supplementary Figure 9 | Quantile-quantile plot of -$\log_{10}$ p-values for PTSD, comparing each class of MBSVs with non-MBSV brain-expressed 3’UTR variants.

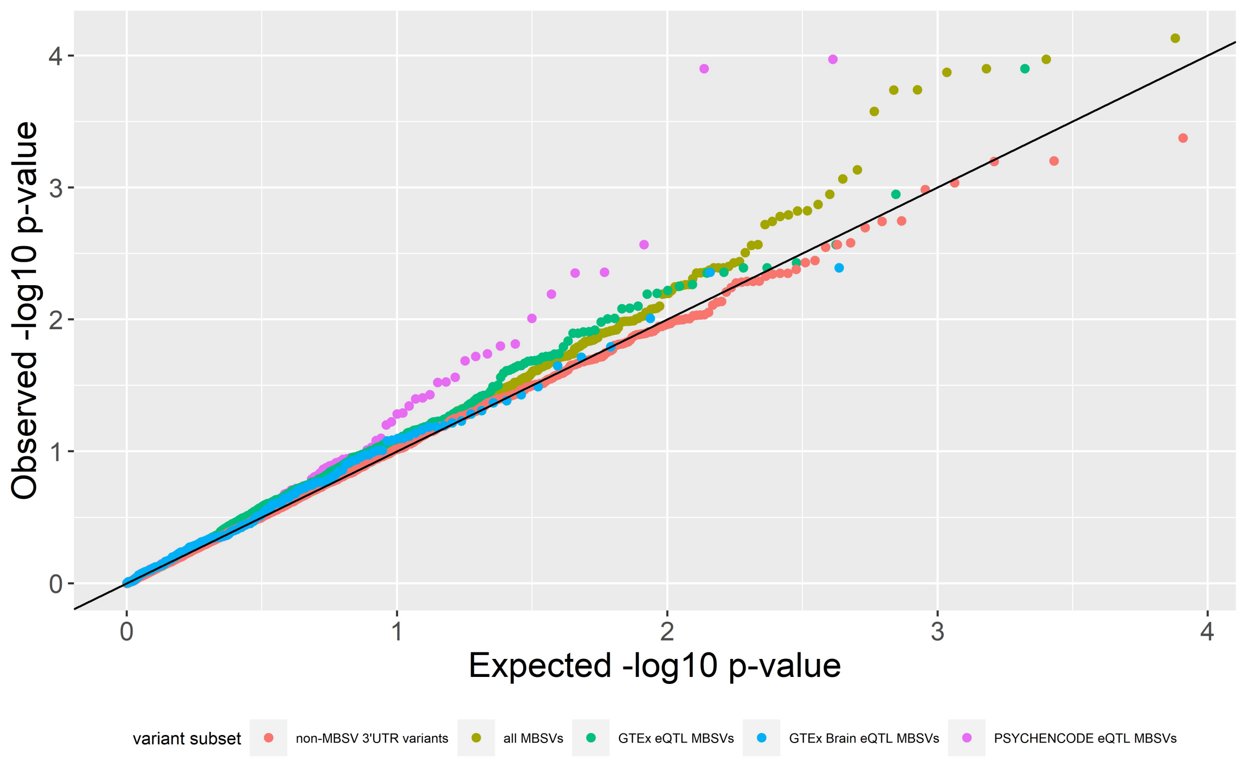

Supplementary Figure 10 | Quantile-quantile plot of -log_{10} p-values for TS, comparing each class of MBSVs with non-MBSV brain-expressed 3’UTR variants.

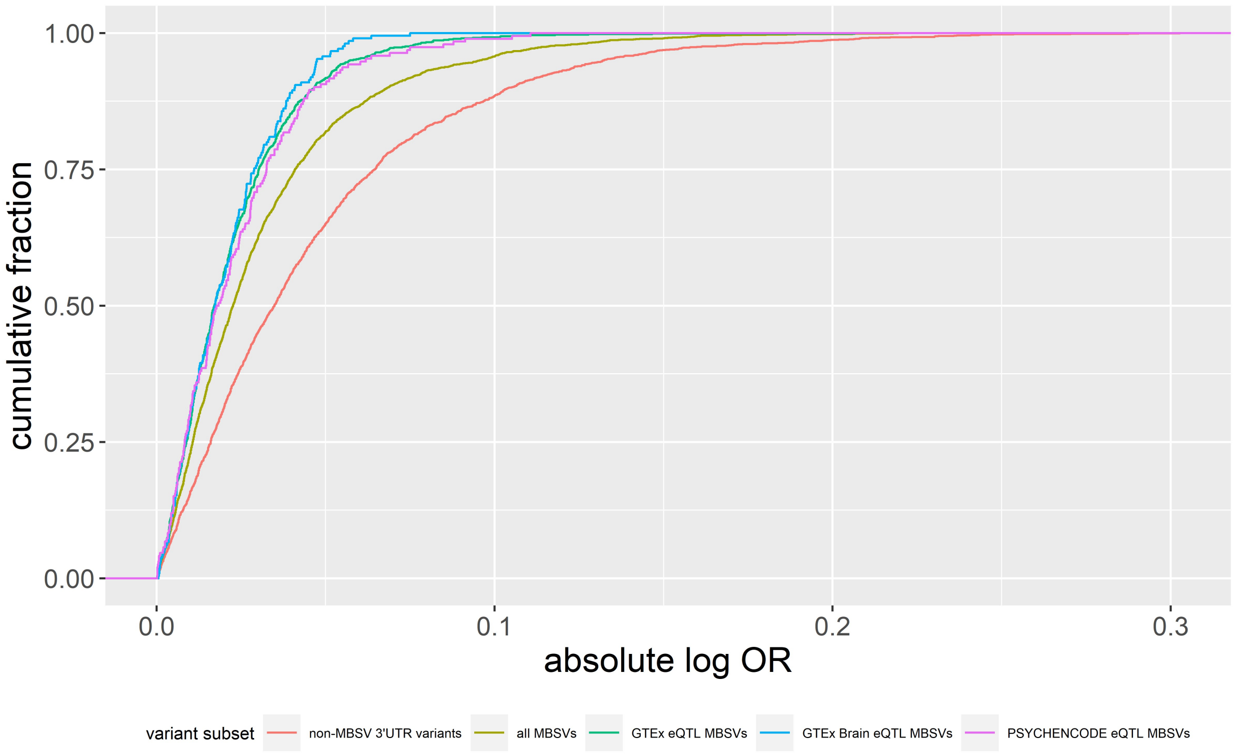

Supplementary Figure 11 | Distribution of absolute log-transformed effect sizes for MBSVs, eQTL MBSVs, and brain-expressed 3'UTR non-MBSVs for ADHD.

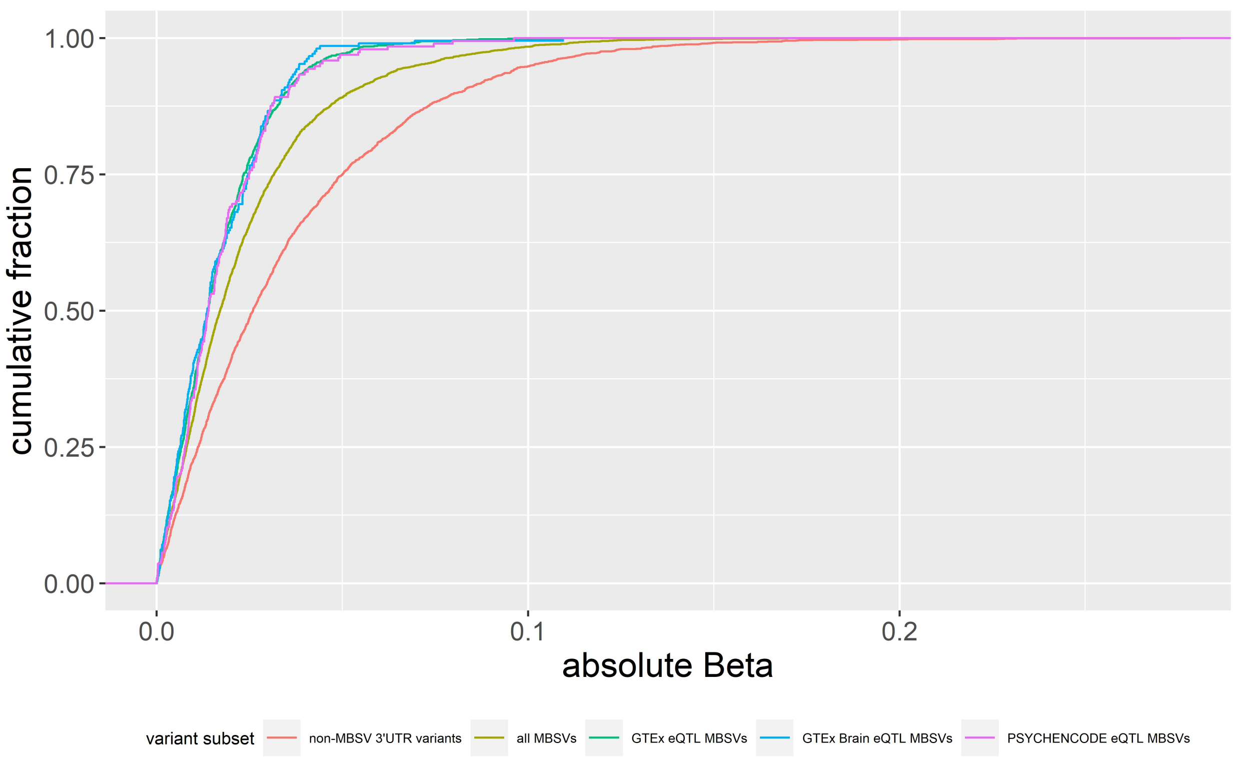

Supplementary Figure 12 | Distribution of absolute log-transformed effect sizes for MBSVs, eQTL MBSVs, and brain-expressed 3’UTR non-MBSVs for AN.

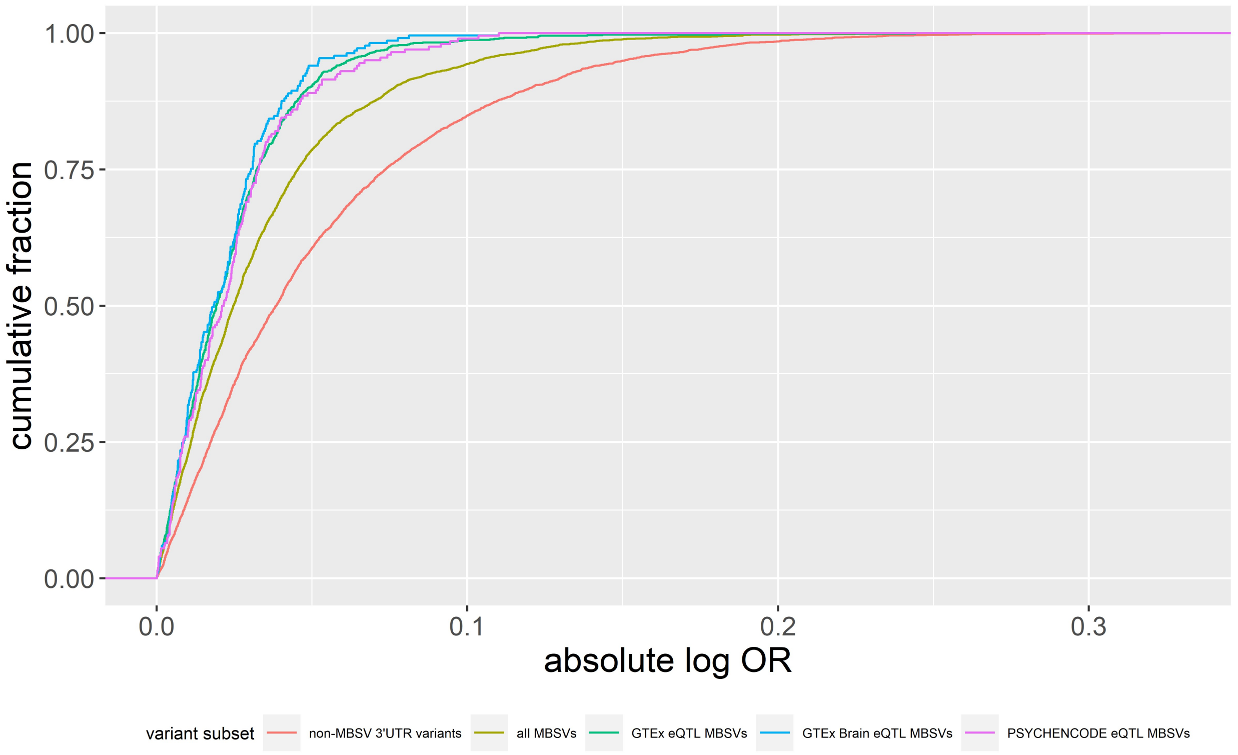

Supplementary Figure 13 | Distribution of absolute log-transformed effect sizes for MBSVs, eQTL MBSVs, and brain-expressed 3’UTR non-MBSVs for ASD.

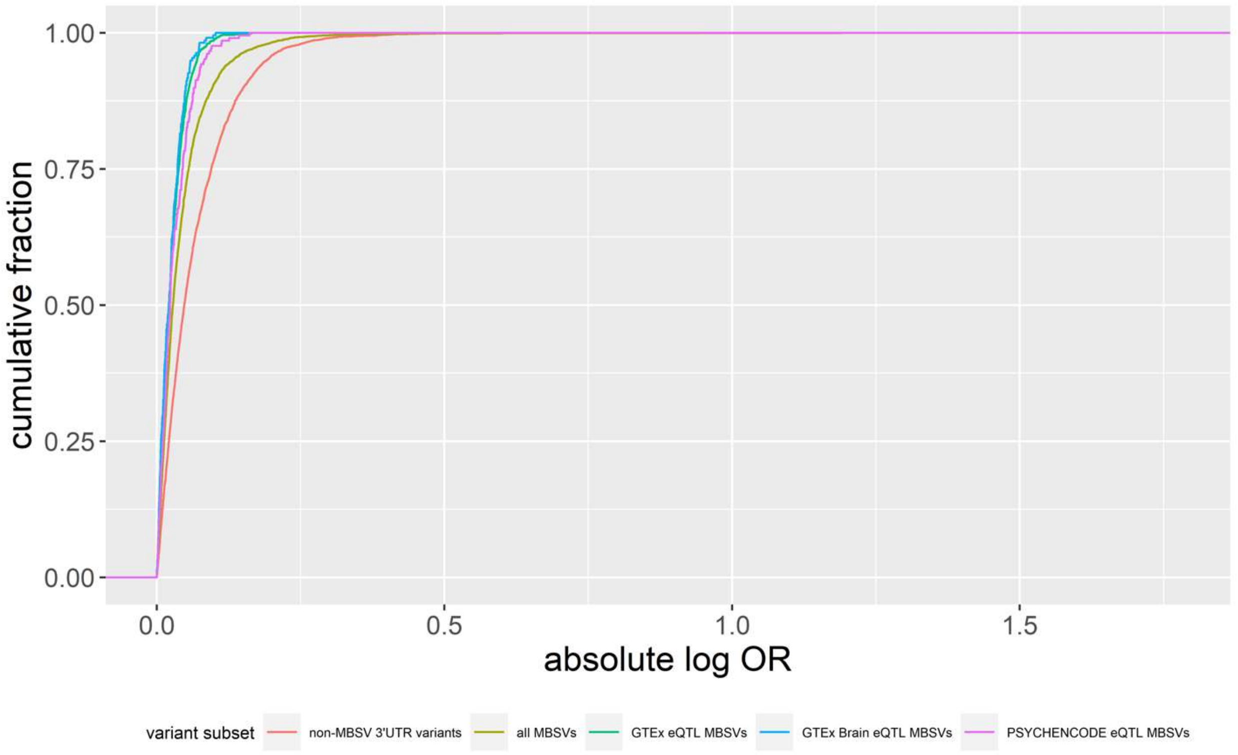

Supplementary Figure 14 | Distribution of absolute log-transformed effect sizes for MBSVs, eQTL MBSVs, and brain-expressed 3'UTR non-MBSVs for BIP.

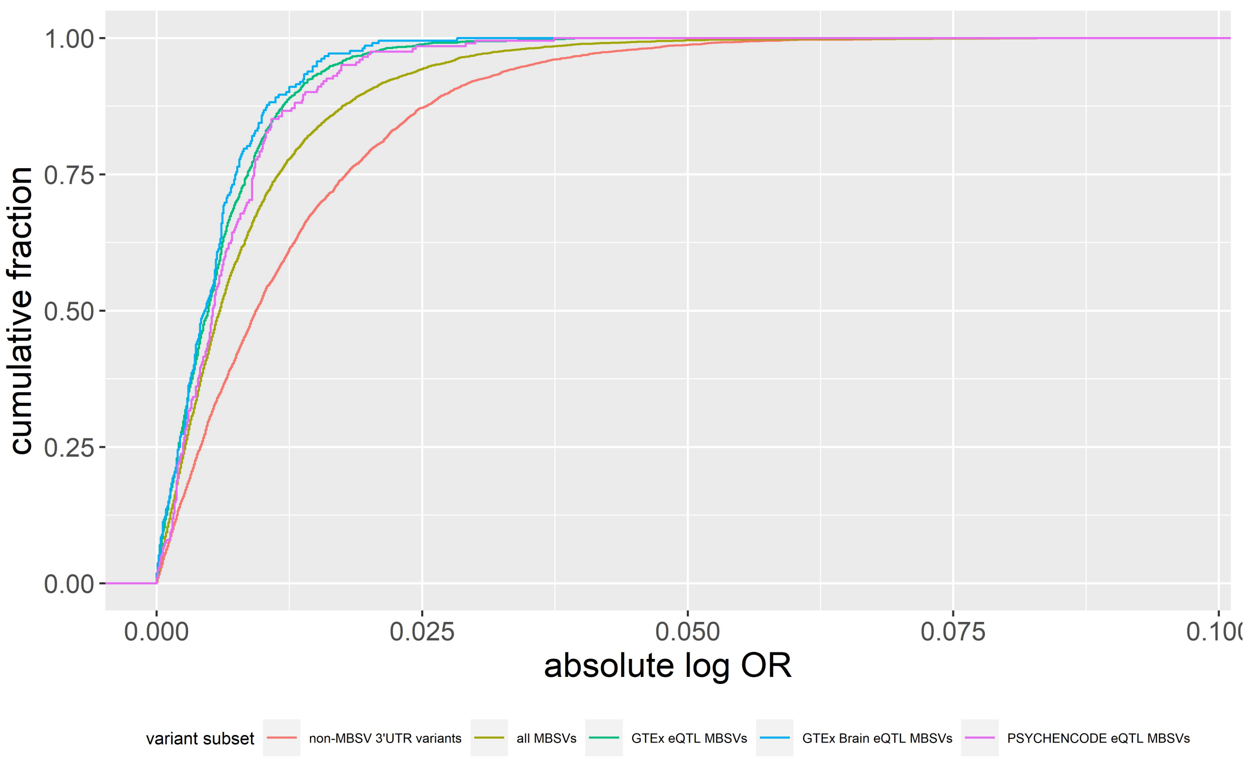

Supplementary Figure 15 | Distribution of absolute log-transformed effect sizes for MBSVs, eQTL MBSVs, and brain-expressed 3’UTR non-MBSVs for MDD.

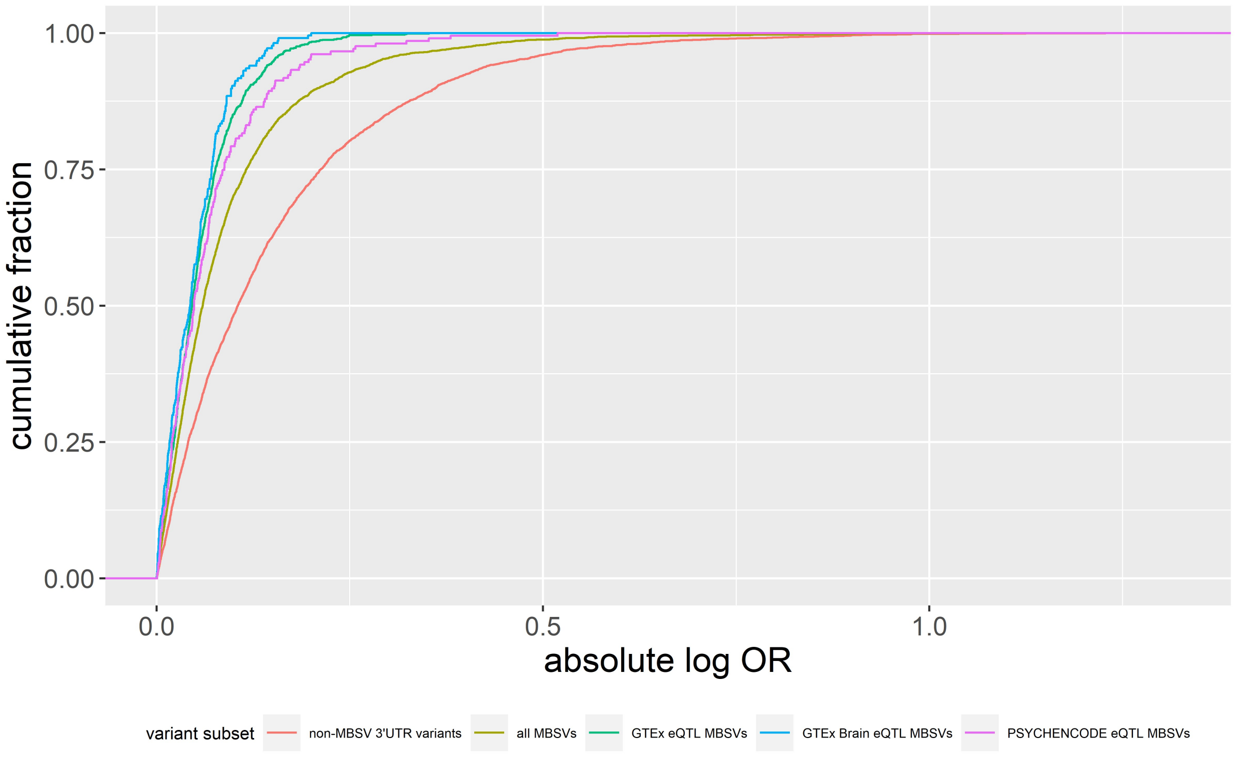

Supplementary Figure 16 | Distribution of absolute log-transformed effect sizes for MBSVs, eQTL MBSVs, and brain-expressed 3’UTR non-MBSVs for OCD.

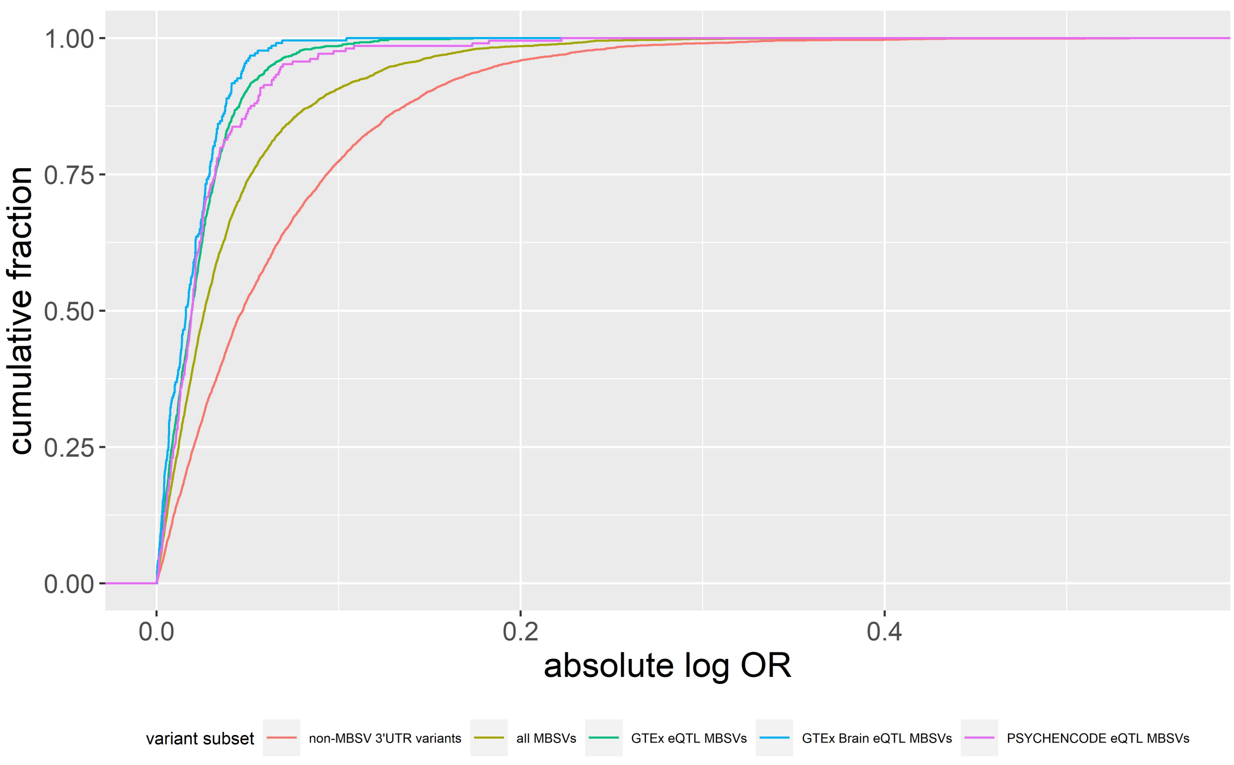

Supplementary Figure 17 | Distribution of absolute log-transformed effect sizes for MBSVs, eQTL MBSVs, and brain-expressed 3'UTR non-MBSVs for PTSD.

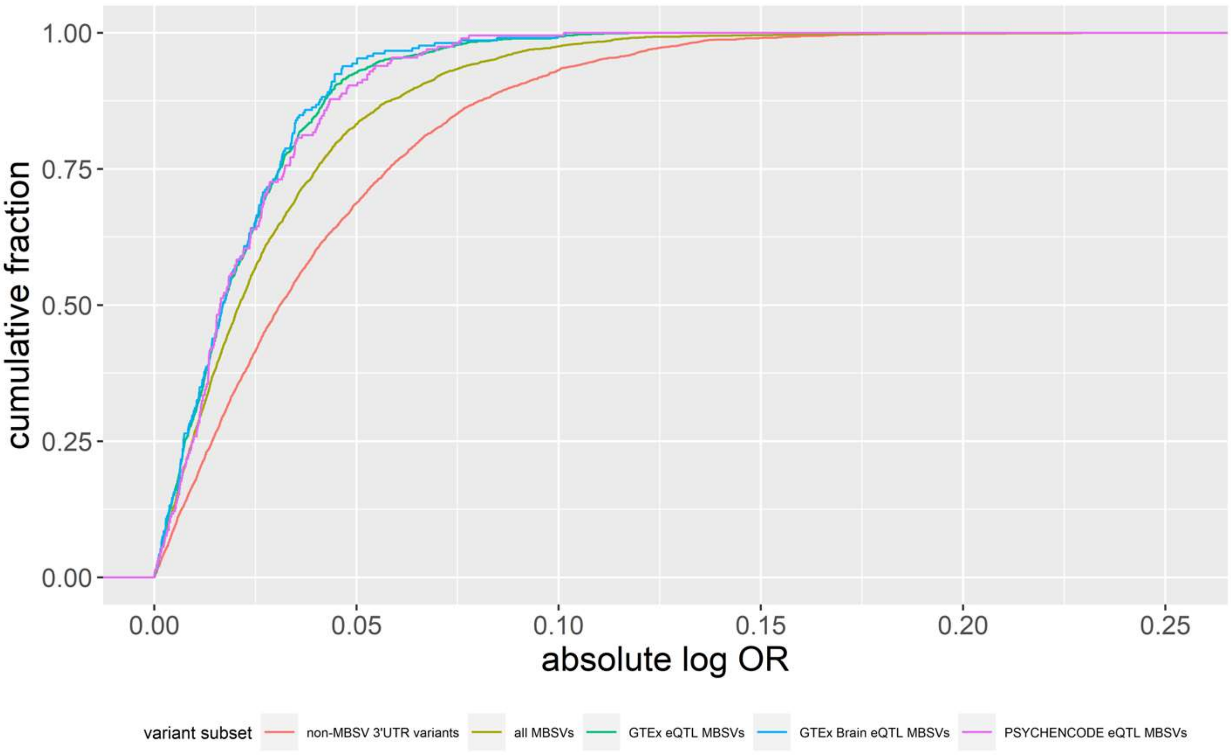

Supplementary Figure 18 | Distribution of absolute log-transformed effect sizes for MBSVs, eQTL MBSVs, and brain-expressed 3'UTR non-MBSVs for SCZ.

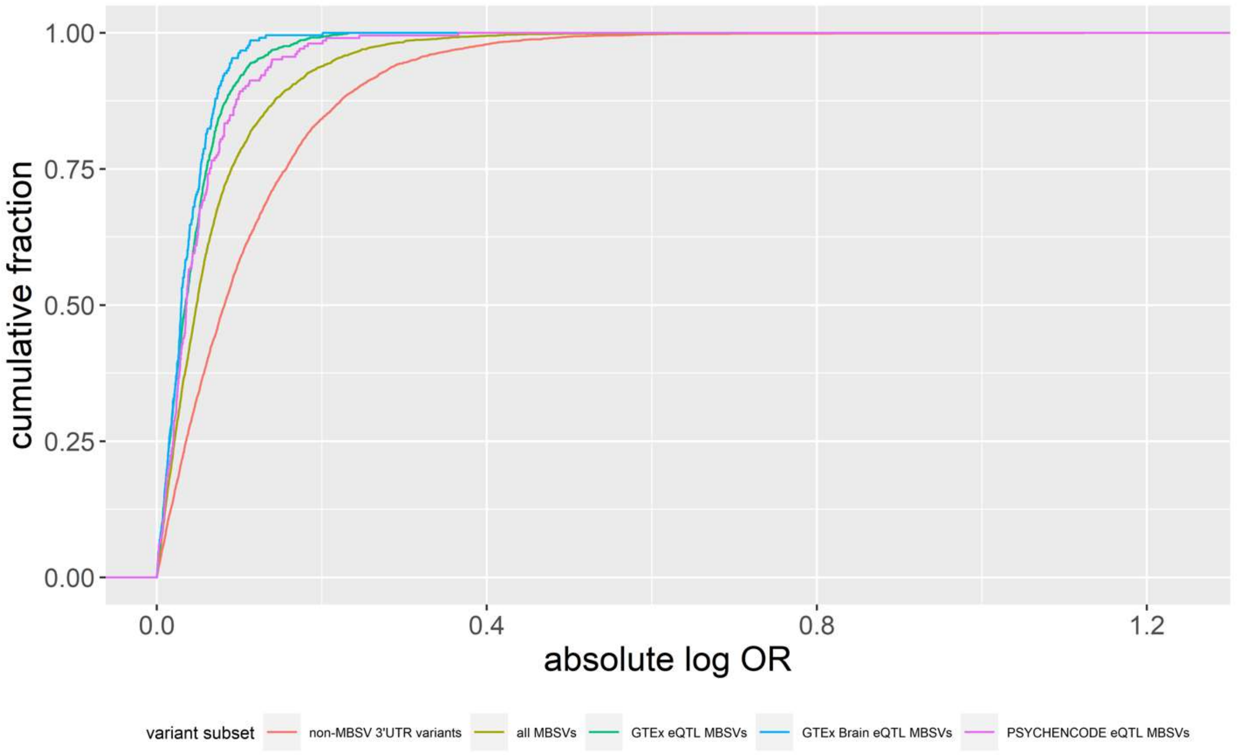

Supplementary Figure 19 | Distribution of absolute log-transformed effect sizes for MBSVs, eQTL MBSVs, and brain-expressed 3’UTR non-MBSVs for TS.

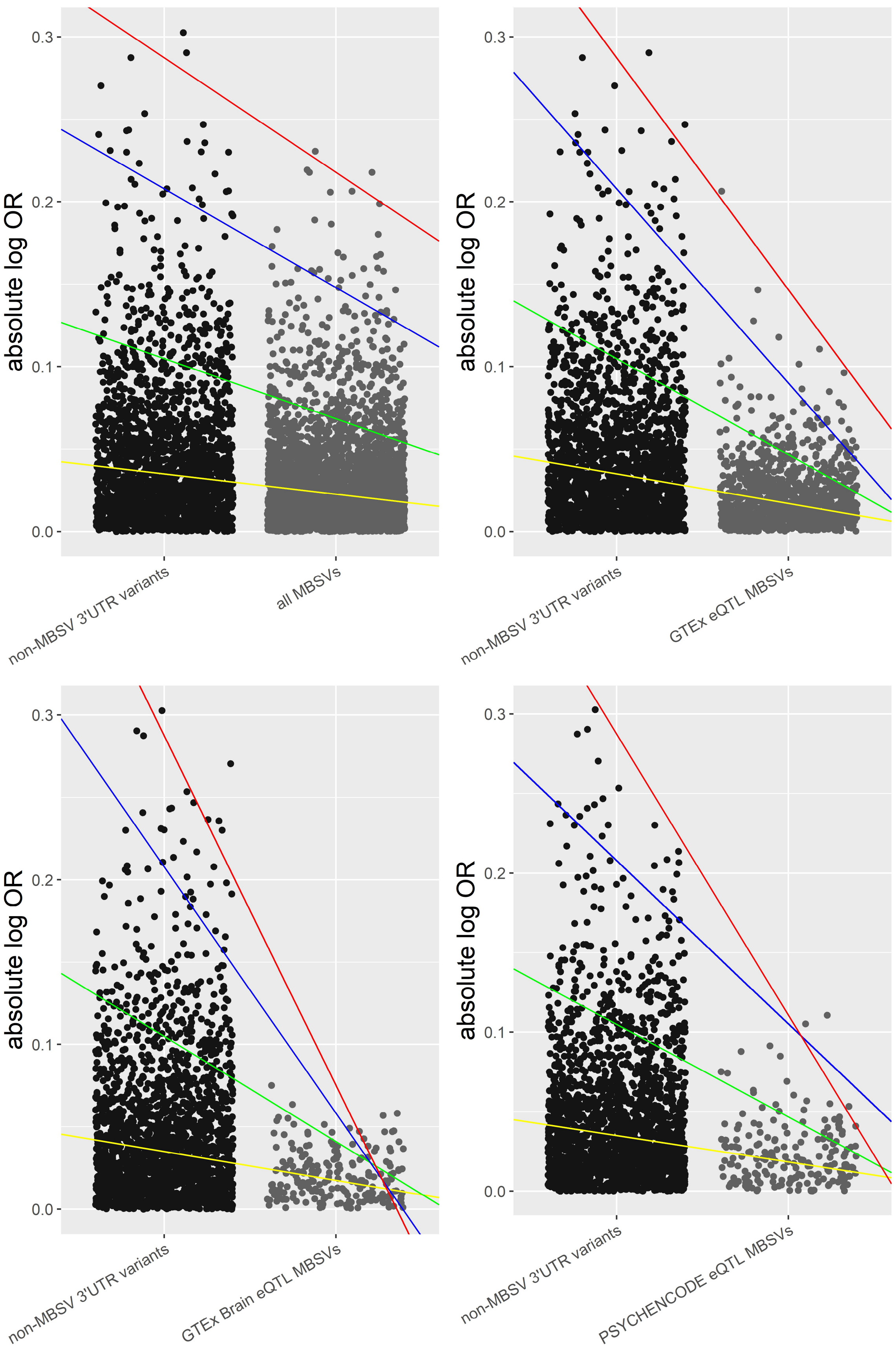

absolute log OR
0.0
0.1
0.2
0.3
non-MBSV 3'UTR variants
all MBSVs
GTEx eQTL MBSVs
GTEx Brain eQTL MBSVs
PSYCHENCODE eQTL MBSVs

Supplementary Figure 20 | Quantile regressions of absolute log-transformed effect sizes for MBSVs, eQTL MBSVs, and brain-expressed 3'UTR non-MBSVs for ADHD. The 50th (median), 90th, 99th, and 99.9^{th} percentiles were compared between brain-expressed 3'UTR non-MBSV variants and either all MBSVs, GTEx eQTL MBSVs, GTEx brain eQTL MBSVs, or PSYCHENCODE eQTL MBSVs. Regression lines: yellow = median, green = 90th percentile, blue = 99th percentile, red = 99.9^{th} percentile.

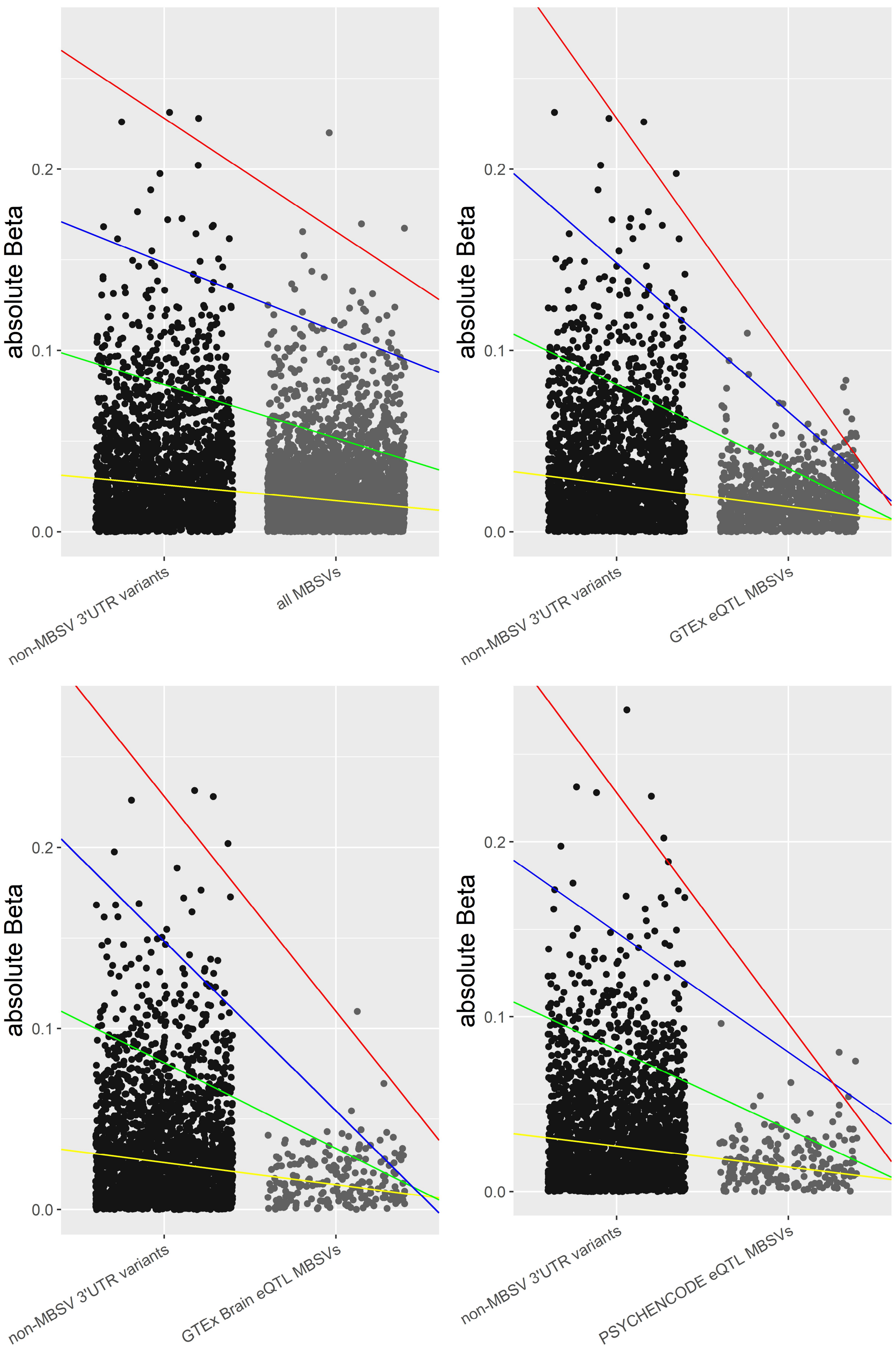

absolute Beta
0.0
0.1
0.2
non-MBSV 3'UTR variants
all MBSVs
GTEx eQTL MBSVs
GTEx Brain eQTL MBSVs
PSYCHENCODE eQTL MBSVs

Supplementary Figure 21 | Quantile regressions of absolute log-transformed effect sizes for MBSVs, eQTL MBSVs, and brain-expressed 3'UTR non-MBSVs for AN. The 50th (median), 90th, 99th, and 99.9th percentiles were compared between brain-expressed 3'UTR non-MBSV variants and either all MBSVs, GTEx eQTL MBSVs, GTEx brain eQTL MBSVs, or PSYCHENCODE eQTL MBSVs. Regression lines: yellow = median, green = 90th percentile, blue = 99th percentile, red = 99.9th percentile.

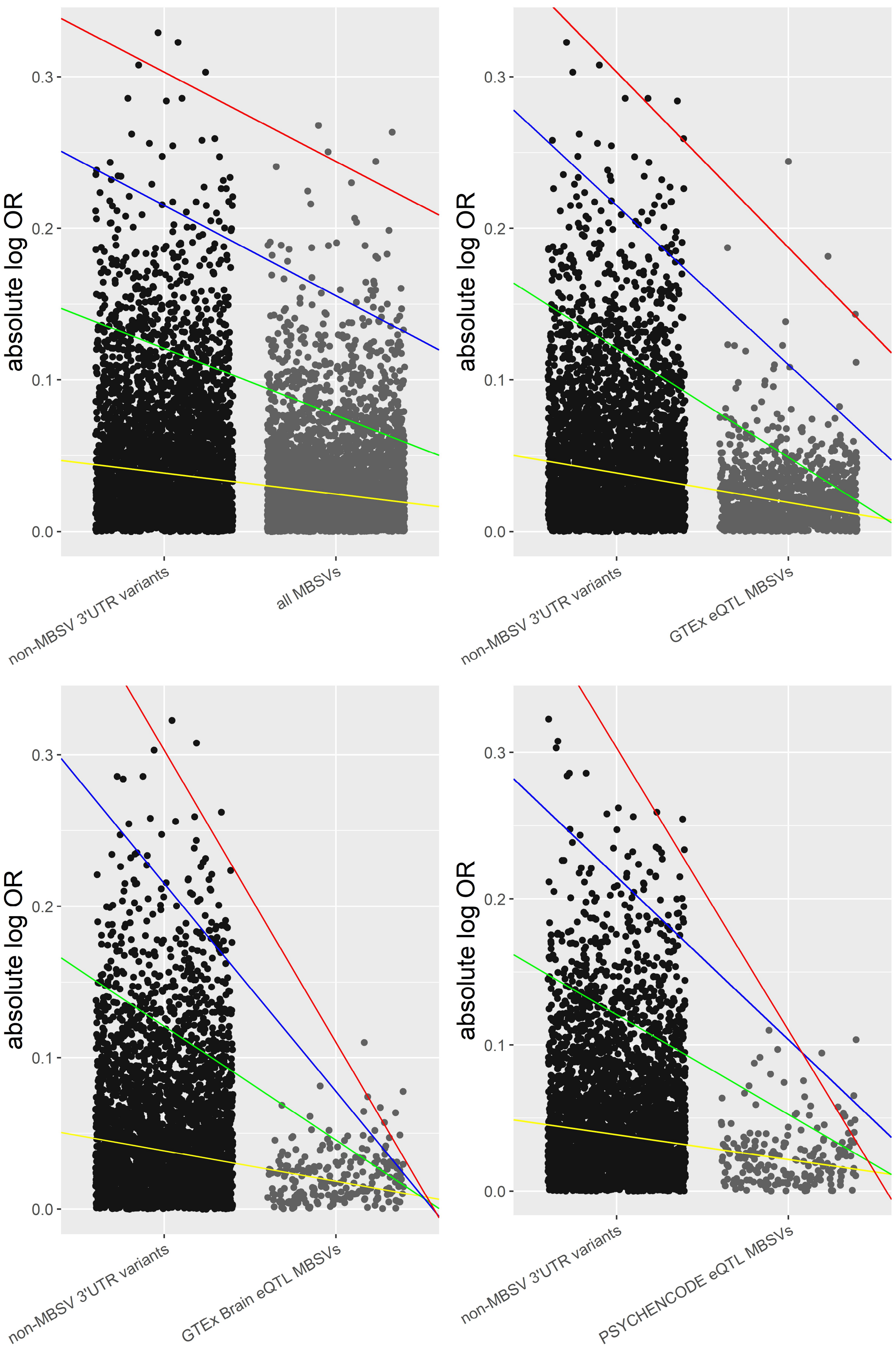
absolute log OR
0.0
0.1
0.2
0.3
non-MBSV 3'UTR variants
all MBSVs
GTEx eQTL MBSVs
GTEx Brain eQTL MBSVs
PSYCHENCODE eQTL MBSVs

Supplementary Figure 22 | Quantile regressions of absolute log-transformed effect sizes for MBSVs, eQTL MBSVs, and brain-expressed 3'UTR non-MBSVs for ASD. The 50th (median), 90th, 99th, and 99.9th percentiles were compared between brain-expressed 3'UTR non-MBSV variants and either all MBSVs, GTEx eQTL MBSVs, GTEx brain eQTL MBSVs, or PSYCHENCODE eQTL MBSVs. Regression lines: yellow = median, green = 90th percentile, blue = 99th percentile, red = 99.9th percentile.

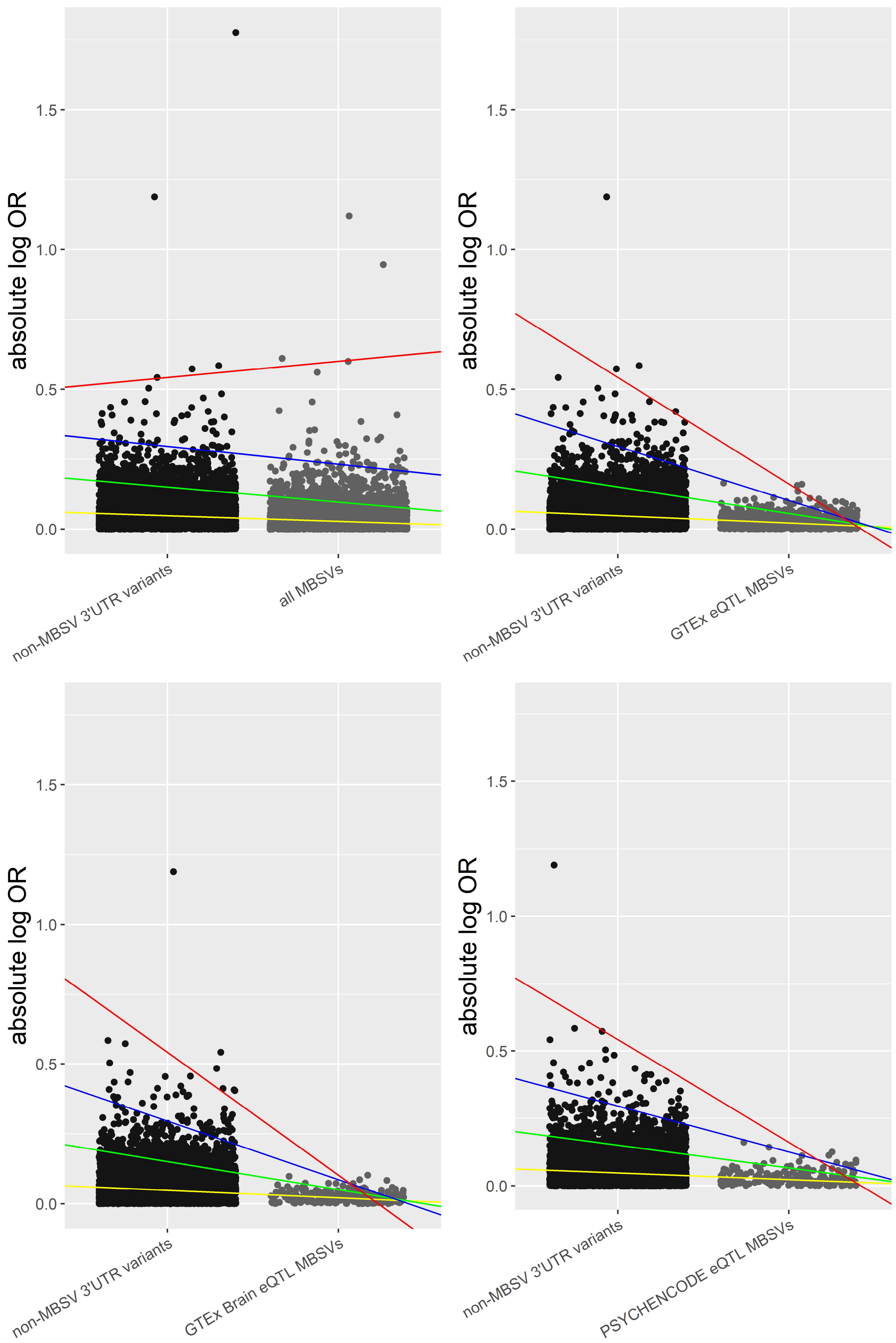
absolute log OR
1.5
1.0
0.5
0.0
non-MBSV 3'UTR variants
all MBSVs
GTEx eQTL MBSVs
GTEx Brain eQTL MBSVs
PSYCHENCODE eQTL MBSVs

Supplementary Figure 23 | Quantile regressions of absolute log-transformed effect sizes for MBSVs, eQTL MBSVs, and brain-expressed 3'UTR non-MBSVs for BIP. The 50th (median), 90th, 99th, and 99.9th percentiles were compared between brain-expressed 3'UTR non-MBSV variants and either all MBSVs, GTEx eQTL MBSVs, GTEx brain eQTL MBSVs, or PSYCHENCODE eQTL MBSVs. Regression lines: yellow = median, green = 90th percentile, blue = 99th percentile, red = 99.9th percentile.

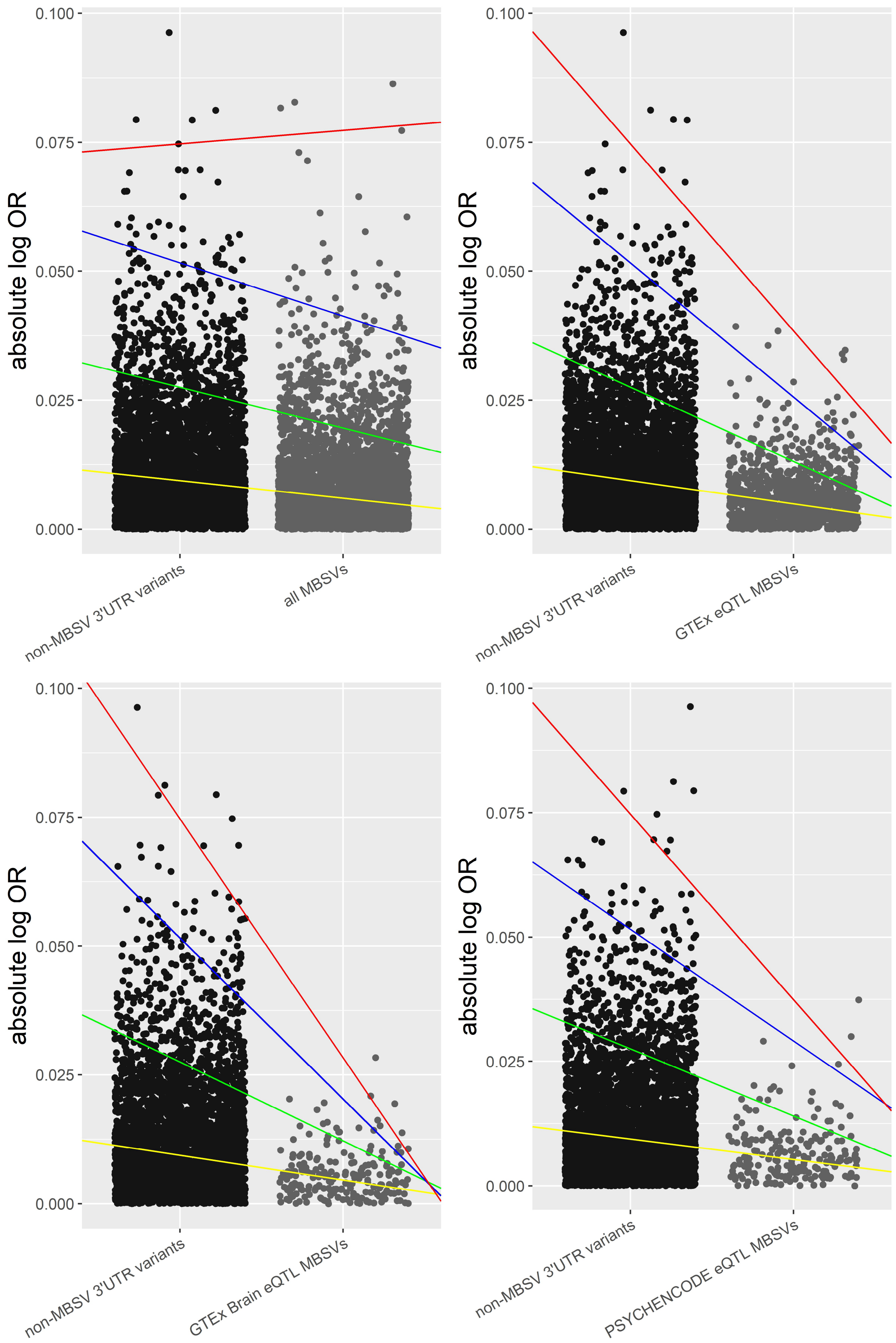

absolute log OR
0.100
0.075
0.050
0.025
0.000
non-MBSV 3'UTR variants
all MBSVs
GTEx eQTL MBSVs
GTEx Brain eQTL MBSVs
PSYCHENCODE eQTL MBSVs

Supplementary Figure 24 | Quantile regressions of absolute log-transformed effect sizes for MBSVs, eQTL MBSVs, and brain-expressed 3'UTR non-MBSVs for MDD. The 50th (median), 90th, 99th, and 99.9th percentiles were compared between brain-expressed 3'UTR non-MBSV variants and either all MBSVs, GTEx eQTL MBSVs, GTEx brain eQTL MBSVs, or PSYCHENCODE eQTL MBSVs. Regression lines: yellow = median, green = 90th percentile, blue = 99th percentile, red = 99.9th percentile.

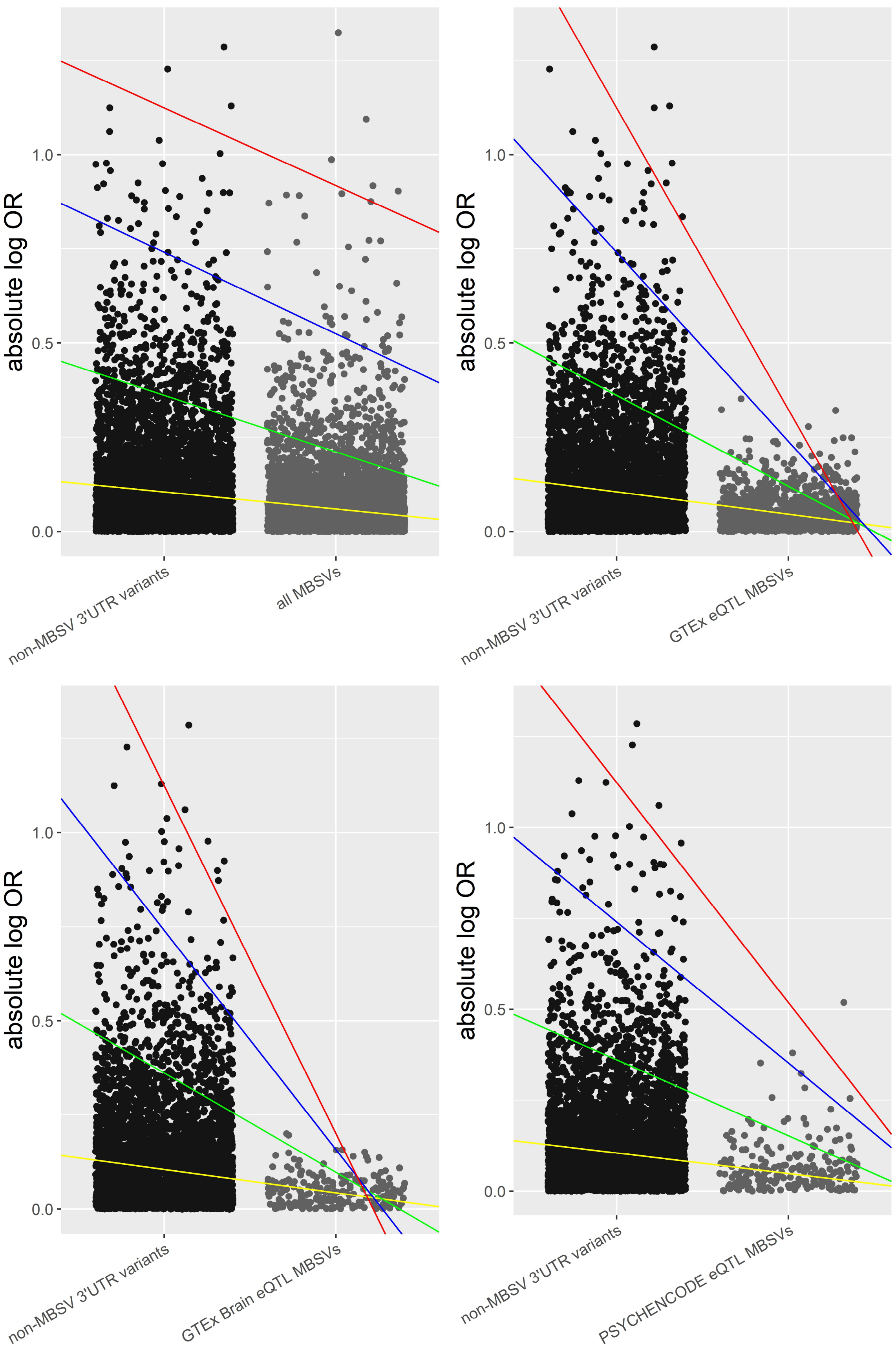
absolute log OR
1.0
0.5
0.0
non-MBSV 3'UTR variants
all MBSVs
GTEx eQTL MBSVs
GTEx Brain eQTL MBSVs
PSYCHENCODE eQTL MBSVs

Supplementary Figure 25 | Quantile regressions of absolute log-transformed effect sizes for MBSVs, eQTL MBSVs, and brain-expressed 3’UTR non-MBSVs for OCD. The 50th (median), 90th, 99th, and 99.9th percentiles were compared between brain-expressed 3’UTR non-MBSV variants and either all MBSVs, GTEx eQTL MBSVs, GTEx brain eQTL MBSVs, or PSYCHENCODE eQTL MBSVs. Regression lines: yellow = median, green = 90th percentile, blue = 99th percentile, red = 99.9th percentile.

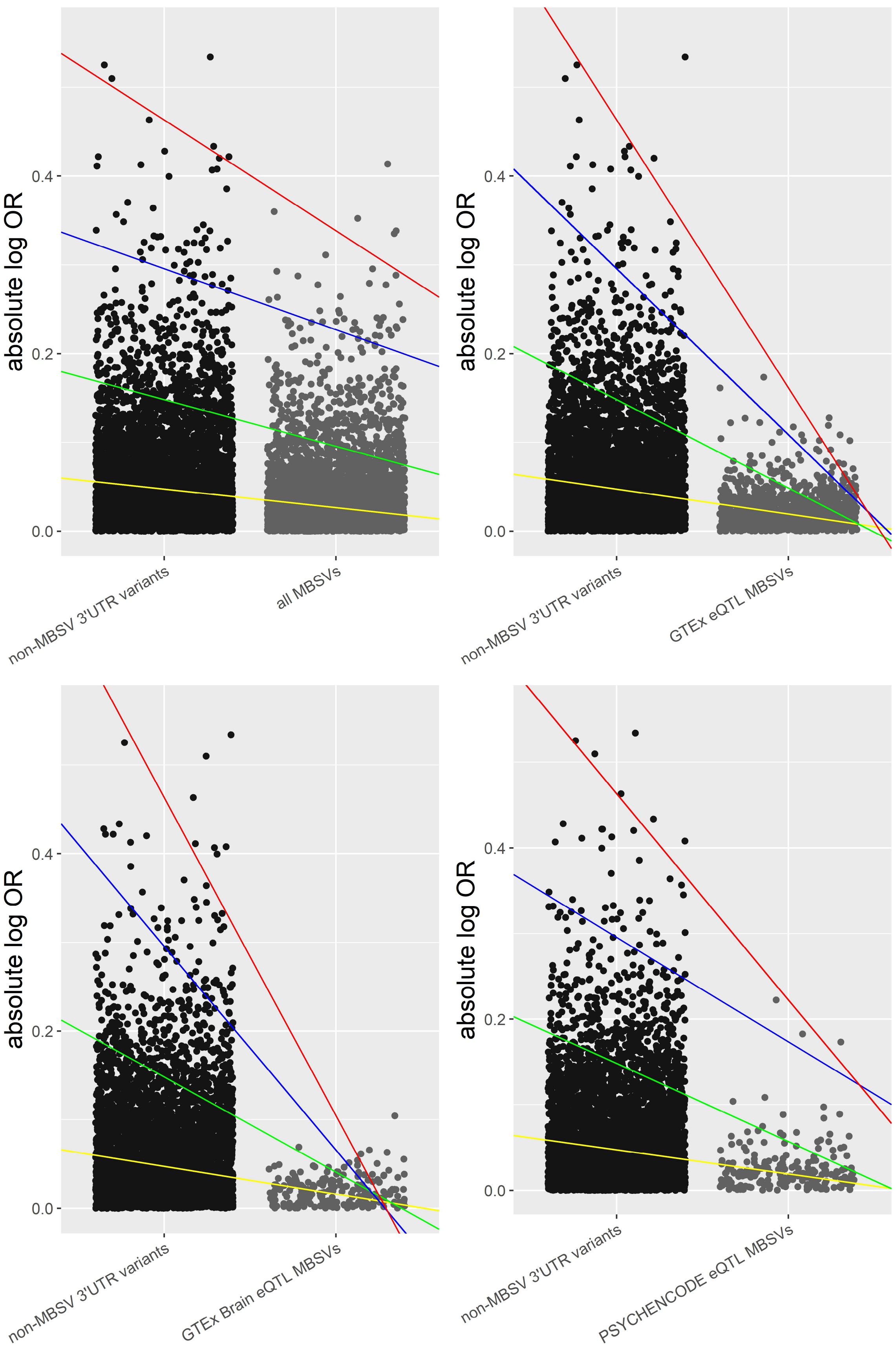

absolute log OR
0.0
0.2
0.4
non-MBSV 3'UTR variants
all MBSVs
GTEx eQTL MBSVs
GTEx Brain eQTL MBSVs
PSYCHENCODE eQTL MBSVs

Supplementary Figure 26 | Quantile regressions of absolute log-transformed effect sizes for MBSVs, eQTL MBSVs, and brain-expressed 3'UTR non-MBSVs for PTSD. The 50th (median), 90th, 99th, and 99.9th percentiles were compared between brain-expressed 3'UTR non-MBSV variants and either all MBSVs, GTEx eQTL MBSVs, GTEx brain eQTL MBSVs, or PSYCHENCODE eQTL MBSVs. Regression lines: yellow = median, green = 90th percentile, blue = 99th percentile, red = 99.9th percentile.

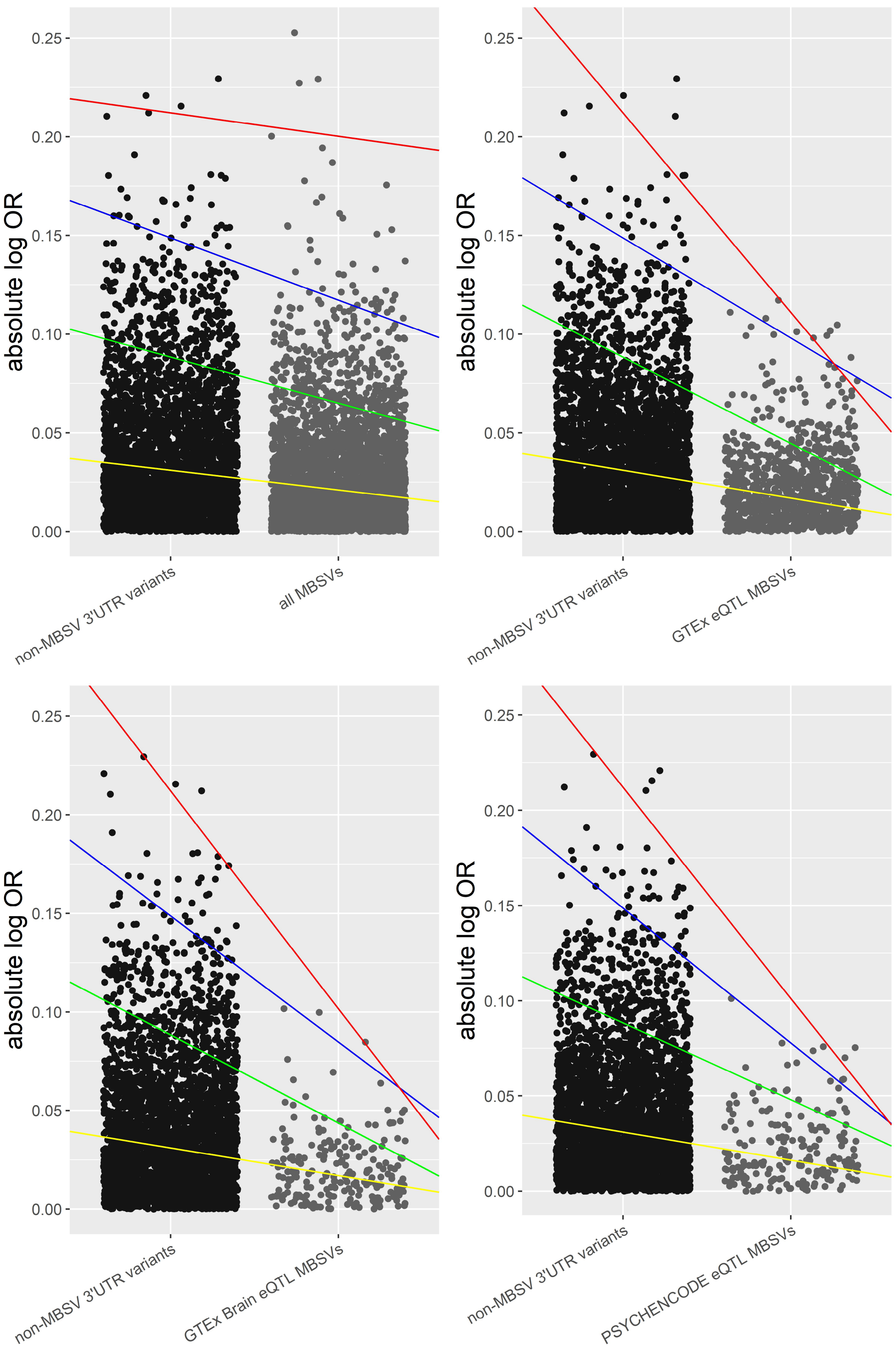
absolute log OR
0.25
0.20
0.15
0.10
0.05
0.00
non-MBSV 3'UTR variants
all MBSVs
GTEx eQTL MBSVs
GTEx Brain eQTL MBSVs
PSYCHENCODE eQTL MBSVs

Supplementary Figure 27 | Quantile regressions of absolute log-transformed effect sizes for MBSVs, eQTL MBSVs, and brain-expressed 3'UTR non-MBSVs for SCZ. The 50th (median), 90th, 99th, and 99.9th percentiles were compared between brain-expressed 3'UTR non-MBSV variants and either all MBSVs, GTEx eQTL MBSVs, GTEx brain eQTL MBSVs, or PSYCHENCODE eQTL MBSVs. Regression lines: yellow = median, green = 90th percentile, blue = 99th percentile, red = 99.9th percentile.

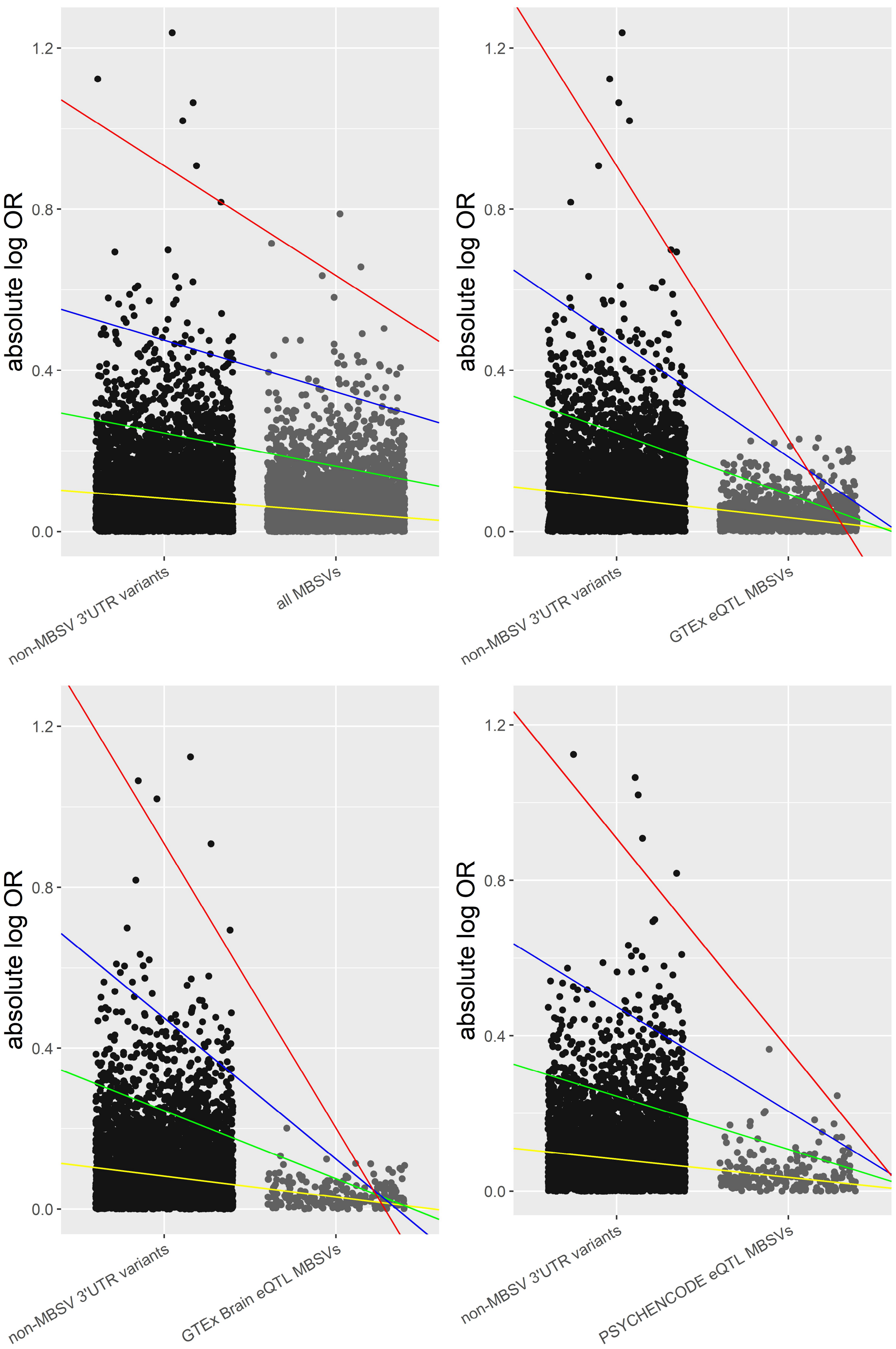
absolute log OR
1.2
0.8
0.4
0.0
non-MBSV 3'UTR variants
all MBSVs
GTEx eQTL MBSVs
GTEx Brain eQTL MBSVs
PSYCHENCODE eQTL MBSVs

Supplementary Figure 28 | Quantile regressions of absolute log-transformed effect sizes for MBSVs, eQTL MBSVs, and brain-expressed 3'UTR non-MBSVs for TS. The 50th (median), 90th, 99th, and 99.9th percentiles were compared between brain-expressed 3'UTR non-MBSV variants and either all MBSVs, GTEx eQTL MBSVs, GTEx brain eQTL MBSVs, or PSYCHENCODE eQTL MBSVs. Regression lines: yellow = median, green = 90th percentile, blue = 99th percentile, red = 99.9th percentile.

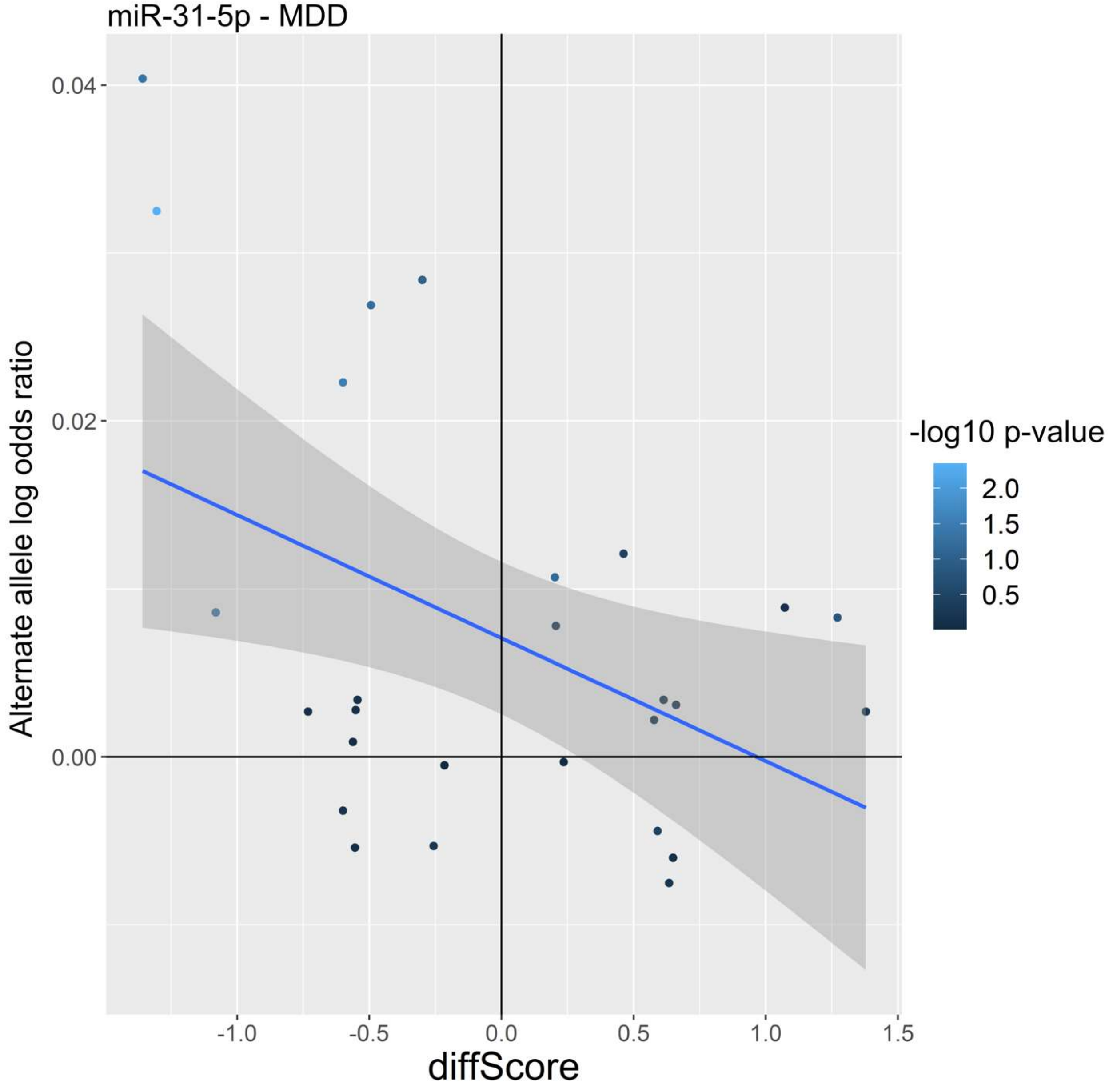

Supplementary Figure 29 | Linear regression analysis investigating the association between the predicted effect of miR-31-5p MBSVs on the miRNA binding site affinities and the log-transformed odds ratios for MDD. This relationship was only nominally significant (p = 0.021, FDR = 0.614).

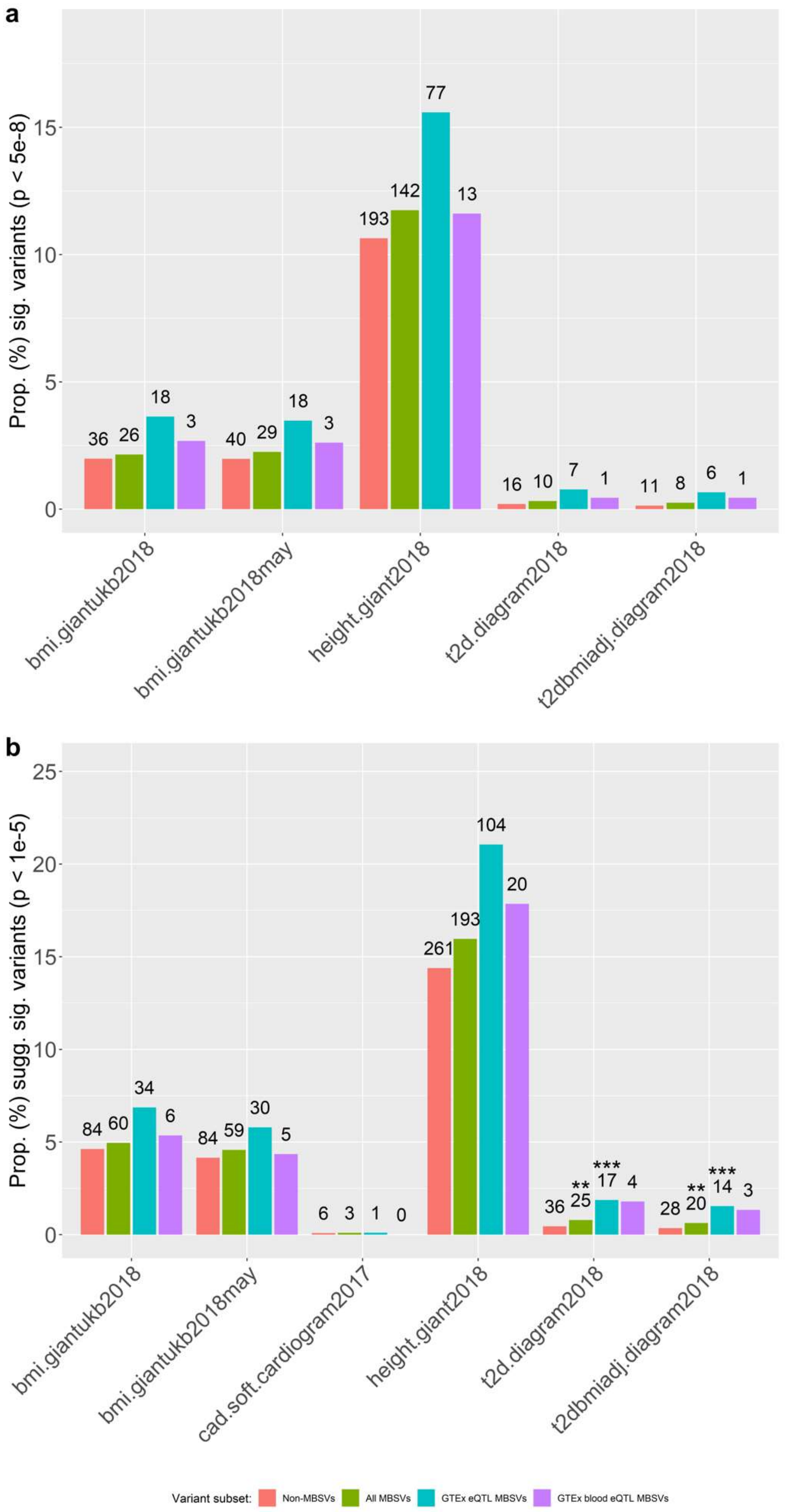
a
Prop. (%) sig. variants (p < 5e-8)
0
5
10
15
36
26
18
3
40
29
18
3
193
142
77
13
16
10
7
1
11
8
6
1
bmi.giantukb2018
bmi.giantukb2018may
height.giant2018
t2d.diagram2018
t2dbmiadj.diagram2018
b
Prop. (%) sugg. sig. variants (p < 1e-5)
0
5
10
15
20
25
84
60
34
6
84
59
30
5
6
3
1
0
261
193
104
20
36
**
25
***
17
4
28
**
20
***
14
3
bmi.giantukb2018
bmi.giantukb2018may
cad.soft.cardiogram2017
height.giant2018
t2d.diagram2018
t2dbmiadj.diagram2018
Variant subset:
Non-MBSVs
All MBSVs
GTEx eQTL MBSVs
GTEx blood eQTL MBSVs

Supplementary Figure 30 | Proportions of strongly-associated variants in each non-psychiatric trait, with MBSVs determined relative to blood gene and miRNA expression. Variants were filtered for genome-wide significance ($p < 5x10^{-8}$) (a) or suggestive significance ($p < 1x10^{-5}$) (b). Proportions of all strongly-associated MBSVs (green) and eQTL-annotated MBSVs (GTEx in blue; GTEx blood in purple) were compared to the proportion of strongly-associated non-MBSVs (red) using Fisher's exact test. Only traits with at least one significant MBSV are shown. Numbers represent counts of significant variants. * = $p < 0.05$; ** = $p < 0.01$; *** = $p < 0.001$.

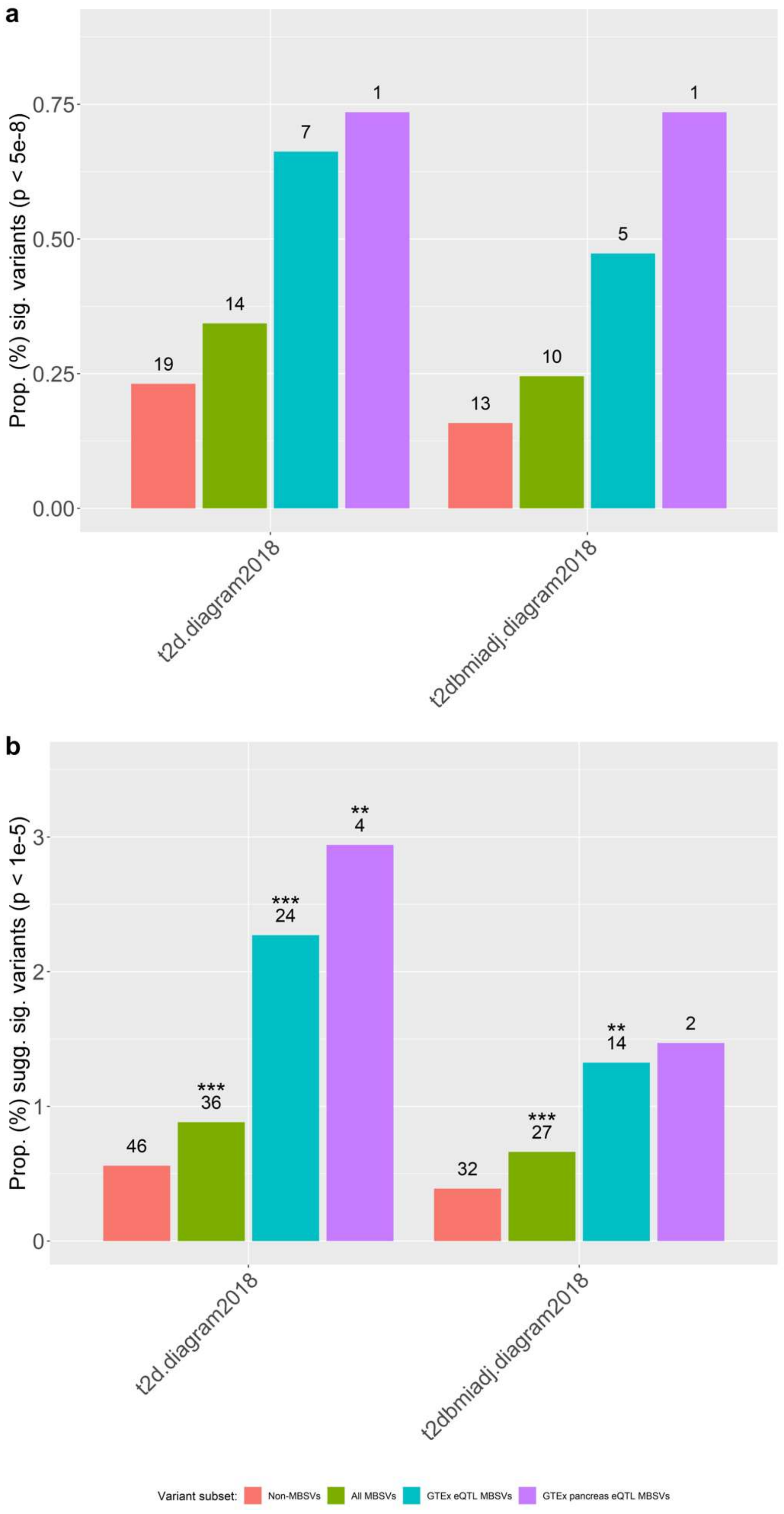

a
Prop. (%) sig. variants (p < 5e-8)
0.00
0.25
0.50
0.75
19
14
7
1
13
10
5
1
t2d.diagram2018
t2dbmiadj.diagram2018
b
Prop. (%) sugg. sig. variants (p < 1e-5)
0
1
2
3
46
***
36
***
24
**
4
32
***
27
**
14
2
t2d.diagram2018
t2dbmiadj.diagram2018
Variant subset:
Non-MBSVs
All MBSVs
GTEx eQTL MBSVs
GTEx pancreas eQTL MBSVs

Supplementary Figure 31 | Proportions of strongly-associated variants in type-2 diabetes, with MBSVs determined relative to pancreas gene and miRNA expression. Variants were filtered for genome-wide significance ($p < 5x10^{-8}$) (a) or suggestive significance ($p < 1x10^{-5}$) (b). Proportions of all strongly-associated MBSVs (green) and eQTL-annotated MBSVs (GTEx in blue; GTEx blood in purple) were compared to the proportion of strongly-associated 3'UTR-localised non-MBSVs (red) using Fisher's exact test. Only traits with at least one significant MBSV are shown. Numbers represent counts of significant variants. * = $p < 0.05$; ** = $p < 0.01$; *** = $p < 0.001$.
